# The FoxO4/DKK3 axis represses IFN-**γ** expression by Th1 cells and limits antimicrobial immunity

**DOI:** 10.1172/JCI147566

**Published:** 2022-09-15

**Authors:** Xiang Chen, Jia Hu, Yunfei Wang, Younghee Lee, Xiaohong Zhao, Huiping Lu, Gengzhen Zhu, Hui Wang, Yu Jiang, Fan Liu, Yongzhen Chen, Byung-Seok Kim, Qinghua Zhou, Xindong Liu, Xiaohu Wang, Seon Hee Chang, Chen Dong

**Affiliations:** 1 Institute for Immunology and School of Medicine, Tsinghua University, Beijing, China.; 2 Department of Immunology, The University of Texas MD Anderson Cancer Center, Houston, Texas, USA.; 3 Lung Cancer Center, West China Hospital, Sichuan University, Chengdu, Sichuan, China.; 4 Department of Systems Biology, and; 5 Department of Melanoma Medical Oncology, The University of Texas MD Anderson Cancer Center, Houston, Texas, USA.; 6 Annoroad Gene Technology Co. Ltd., Beijing, China.; 7 Jiangsu Key Laboratory of Immunity and Metabolism, Department of Pathogenic Biology and Immunology, Xuzhou Medical University, Xuzhou, Jiangsu, China.; 8 Faculty of Dentistry, The University of Hong Kong, Hong Kong SAR, China.; 9 Institute of Pathology and Southwest Cancer Center, Southwest Hospital, Third Military Medical University, Chongqing, China.; 10 Shanghai Immune Therapy Institute, Shanghai Jiao Tong University School of Medicine-Affiliated Renji Hospital, Shanghai, China.

**Keywords:** Immunology, Bacterial infections, Cytokines, Th1 response

## Abstract

Forkhead box O transcriptional factors, especially FoxO1 and FoxO3a, play critical roles in physiologic and pathologic immune responses. However, the function of FoxO4, another main member of the FoxO family, in lymphoid cells is still poorly understood. Here, we showed that loss of *FoxO4* in T cells augmented IFN-γ production of Th1 cells in vitro. Correspondingly, conditional deletion of *FoxO4* in CD4^+^ T cells enhanced T cell–specific responses to *Listeria monocytogenes* infection in vivo. Genome-wide occupancy and transcriptomic analyses identified *Dkk3* (encoding the Dickkopf-3 protein) as a direct transcriptional target of FoxO4. Consistent with the FoxO4-DKK3 relationship, recombinant DKK3 protein restored normal levels of IFN-γ production in *FoxO4*-deficient Th1 cells through the downregulation of lymphoid enhancer–binding factor 1 (*Lef1*) expression. Together, our data suggest a potential FoxO4/DKK3 axis in Th1 cell differentiation, providing what we believe to be an important insight and supplement for FoxO family proteins in T lymphocyte biology and revealing a promising target for the treatment of immune-related diseases.

## Graphical abstract

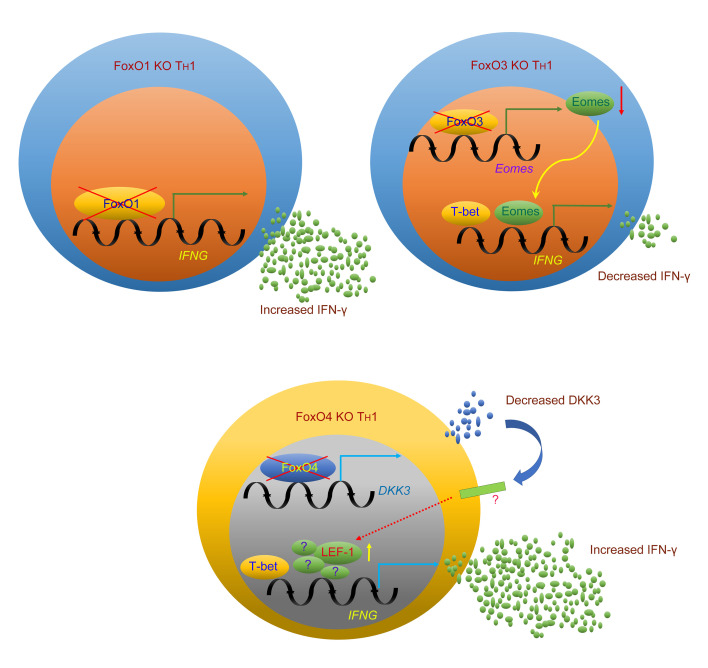

## Introduction

CD4^+^ helper T cells are central regulators of adaptive immune responses. After encountering a specific antigen on antigen-presenting cells, CD4^+^ T cells undergo clonal expansion and differentiate into functionally distinct effector subsets, including at least T helper 1 (Th1), Th2, Th17, and T follicular helper (Tfh) cells, which orchestrate immune responses against diverse microbial pathogens. Among these, IFN-γ–producing Th1 cells specialize in activating cell-mediated immune responses against intracellular pathogens and viruses. The differentiation of CD4^+^ T cells into Th1 cells is determined by T-bet (encoded by *Tbx21*), the master regulator of the Th1 differentiation program (1). Initially, T-bet is induced in response to TCR stimulation and IFN-γ/STAT1 signaling (2, 3). T-bet functions, in part, to upregulate the expression of *Il12rb* (encoding IL-12Rβ), enabling developing Th1 cells to respond to IL-12 (4). As a result, a fully polarized Th1 phenotype is established by IL-12–induced STAT4 activation (5). Thus, the T-bet–STAT4 transcriptional regulatory network maintains stability of the Th1 differentiation program, ensuring CD4^+^ T cells receive proinflammatory signals as well as antigen stimulation to go fully committed into the Th1 cell lineage.

FoxO transcription factors belong to the family of forkhead proteins that are characterized by the presence of an approximately 100-residue forkhead DNA-binding domain. FoxO proteins function as transcriptional regulators and activate the transcription of downstream genes involved in a variety of biological processes including cellular metabolism, organ development, stress responses, and apoptosis (6, 7). In lymphoid cells, FoxO1 and FoxO3a have been shown to cooperatively regulate the generation of Foxp3^+^ Tregs from conventional T cells by binding to the promoter and the conserved CNS2 intronic enhancer region of the *Foxp3* locus (8, 9). In addition, FoxO1 inhibits Th1 differentiation through direct binding to the *Ifng* gene promoter region (8). Conversely, FoxO3a drives pathogenic Th1 differentiation by inducing Eomes expression (10). In addition to Tregs and Th1 cells, FoxO transcription factors have also been reported to negatively regulate the generation of Tfh and Th17 cells. Mice with T cell–specific *FoxO1* deletion accumulate a large population of Tfh cells, perhaps because FoxO1 binds to the *Bcl6* gene locus and mediates its transcriptional repression (11). FoxO1 suppresses the Th17 program in vitro and in vivo by blocking RORγt binding to its target genes (12). In addition, FoxO4 has been shown to regulate insulin signaling and apoptosis (13), yet its role in lymphoid cell biology has not been well addressed.

The Dickkopf (DKK) family of glycoproteins (DKK1–4) are involved in modulating Wnt signaling pathways (14). As the best-characterized member of the DKK family, DKK1, a natural inhibitor of Wnt signaling, inhibits tumor growth and metastasis (15) and promotes Th2 differentiation (16). Moreover, DKK2 was reported to promote tumor immunity evasion through a Wnt-independent signaling pathway (17). In contrast to DKK1 and DKK2, the signaling by DKK3 is still unclear, with reports showing no effect, promotion, or inhibition of the Wnt signaling pathway (18–20). Similarly, the functional roles of DKK3 in immunity are unclear, with conflicting studies reporting its immunomodulatory or immunostimulatory functions (21, 22), suggesting that DKK3 may regulate immunity through a different mechanism.

In this study, we investigated the role of FoxO4 in T cells and found that loss of *FoxO4* enhanced IFN-γ production and the effector function of Th1 cells in vitro and in vivo. Mechanistically, we identified *Dkk3* as a direct transcriptional target of FoxO4, which inhibits IFN-γ production through the downregulation of lymphoid enhancer–binding factor 1 (*Lef1*) expression. Thus, our work identifies a critical axis of FoxO4/DKK3/LEF-1 in regulating Th1 cell differentiation, which is different from other FoxO family members.

## Results

### FoxO4 is dispensable in T cell homeostasis.

**Figure 1:**
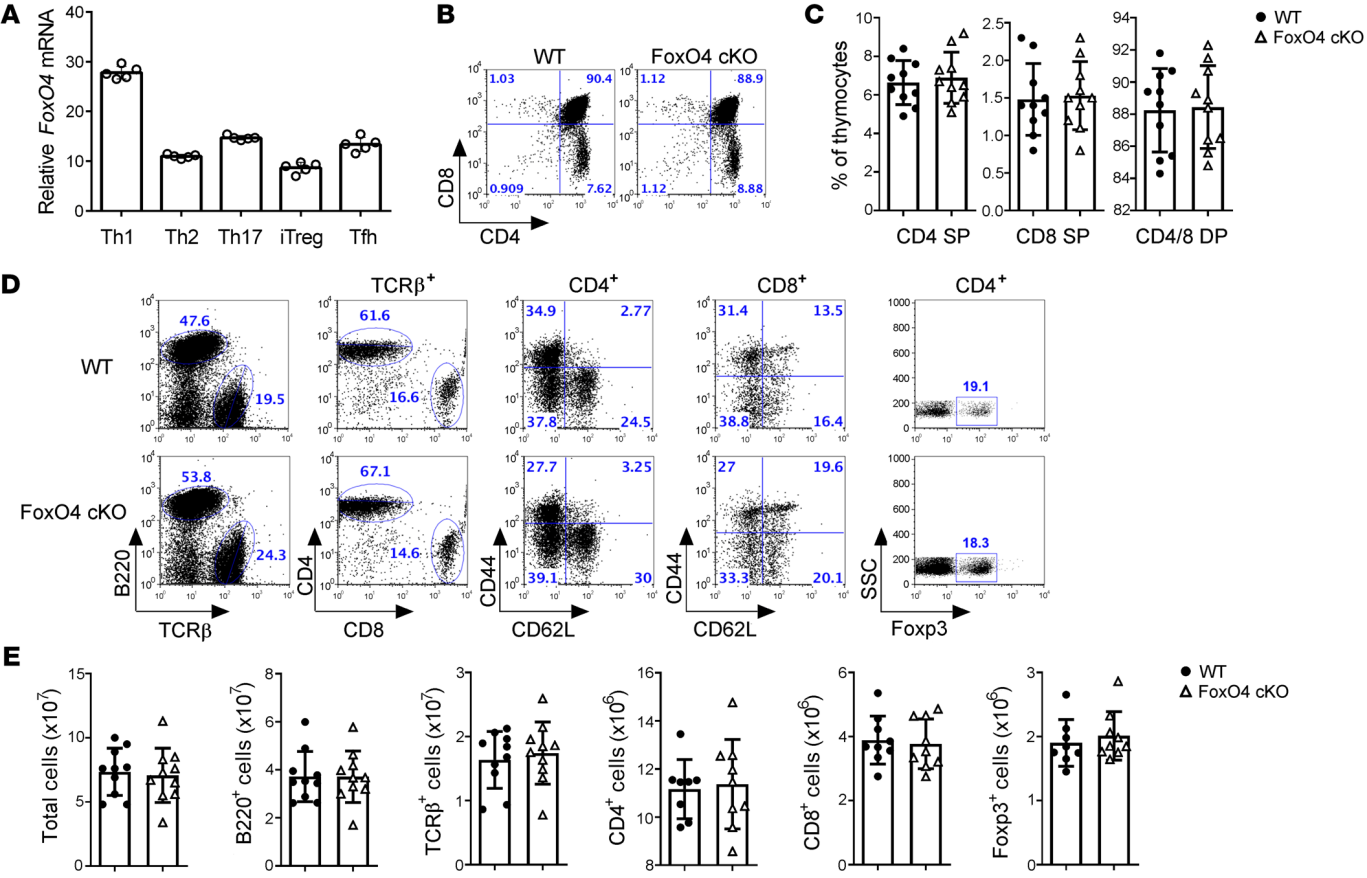
FoxO4 deficiency in CD4^+^ T cells has no apparent effect on T cell homeostasis. (**A**) Real-time qPCR analysis of *FoxO4* mRNA in B6 naive CD4^+^CD44^lo^CD62L^hi^CD25^−^ T cells differentiated for 72 hours in Th1-, Th2-, Th17-, iTreg-, and Tfh-polarizing conditions. Results are presented relative to the expression of *Gapdh* mRNA. (**B**) Flow cytometric analysis of CD4 and CD8 expression in WT and *FoxO4*-cKO thymocytes (*n* = 10). The numbers adjacent to the outlined areas or in the quadrants indicate the percentage of cells. (**C**) Percentages of CD4 SP, CD8 SP, and CD4/8 DP cells in WT and *FoxO4-*cKO thymocytes (*n* = 10). (**D**) Flow cytometric analysis of B220, TCRβ, CD4 (gated on TCRβ^+^), CD8 (gated on TCRβ^+^), CD44 (gated on TCRβ^+^CD4^+^ or TCRβ^+^CD8^+^), CD62L (gated on TCRβ^+^CD4^+^or TCRβ^+^CD8^+^), and Foxp3 (gated on TCRβ^+^CD4^+^) expression on splenocytes isolated from WT and *FoxO4-*cKO mice (*n* = 10). SSC, side scatter. (**E**) Absolute numbers of total cells, B220^+^ B cells, TCRβ^+^ T cells, CD4^+^ T cells, CD8^+^ T cells and Foxp3^+^ T cells in spleens from WT and *FoxO4*-cKO mice (*n* = 10). Each symbol in **C** and **E** represents an individual mouse. NS, by unpaired, 2-tailed Student’s *t* test (**C** and **E**). Data are representative of 3 independent experiments with similar results (mean ±SD in **A**, **C**, and **E**).

To study the role of FoxO4 in T lymphocytes, we first examined its expression in naive CD4^+^ T cells and followed the in vitro differentiation of different T cell subsets. Although Th2, Th17, and induced Tregs (iTregs) exhibited modest levels of *FoxO4* mRNA expression, *FoxO4* expression was substantially higher in Th1 cells differentiated with IL-12 plus IL-2 (Figure 1A), suggesting an important role in Th1 cells. To better investigate the function of FoxO4 in T cells, we generated T cell–specific *FoxO4*-deficient mice (*FoxO4^fl/fl^* *Cd4^Cre^* mice; referred to hereafter as *FoxO4*-cKO) by breeding *FoxO4^fl/fl^* mice (23) with *Cd4^Cre^-*transgenic mice, and their *FoxO4^fl/fl^* littermates (referred to as WT) were used as controls in our studies. *FoxO4*-cKO mice, aged between 6 and 8 weeks, showed normal percentages of CD4^+^ single-positive (CD4 SP), CD8^+^ single-positive (CD8 SP), and CD4^+^CD8^+^ double-positive (CD4/8 DP) thymocytes as well as normal percentages and numbers of TCRβ^+^ cells (Figure 1, B and C, and Supplemental Figure 1, A–C; supplemental material available online with this article; https://doi.org/10.1172/JCI147566DS1), suggesting no major defect in TCR signaling during positive selection in *FoxO4*-cKO mice. Also, spleens from *FoxO4*-cKO mice had percentages and numbers of TCRβ^+^ cells similar to those of WT mouse spleens (Figure 1, D and E). Moreover, *FoxO4*-cKO mice did not exhibit a spontaneous inflammatory phenotype (data not shown). However, unlike *FoxO1-*deficient mice, *FoxO4*-cKO mice had slightly increased percentages and numbers of CD62L^+^ , but not CD44^+^, CD4^+^, or CD8^+^ T cells isolated from the spleens (Figure 1, D and E). In addition, CD4^+^Foxp3^+^ Tregs in spleens from *FoxO4*-cKO mice were present at percentages and numbers similar to those in WT mice (Figure 1, D and E). Thus, T cell development and homeostasis remained normal in T cell–specific *FoxO4*-deficient mice.

### FoxO4 negatively regulates IFN-γ production in Th1 cells in vitro.

**Figure 2:**
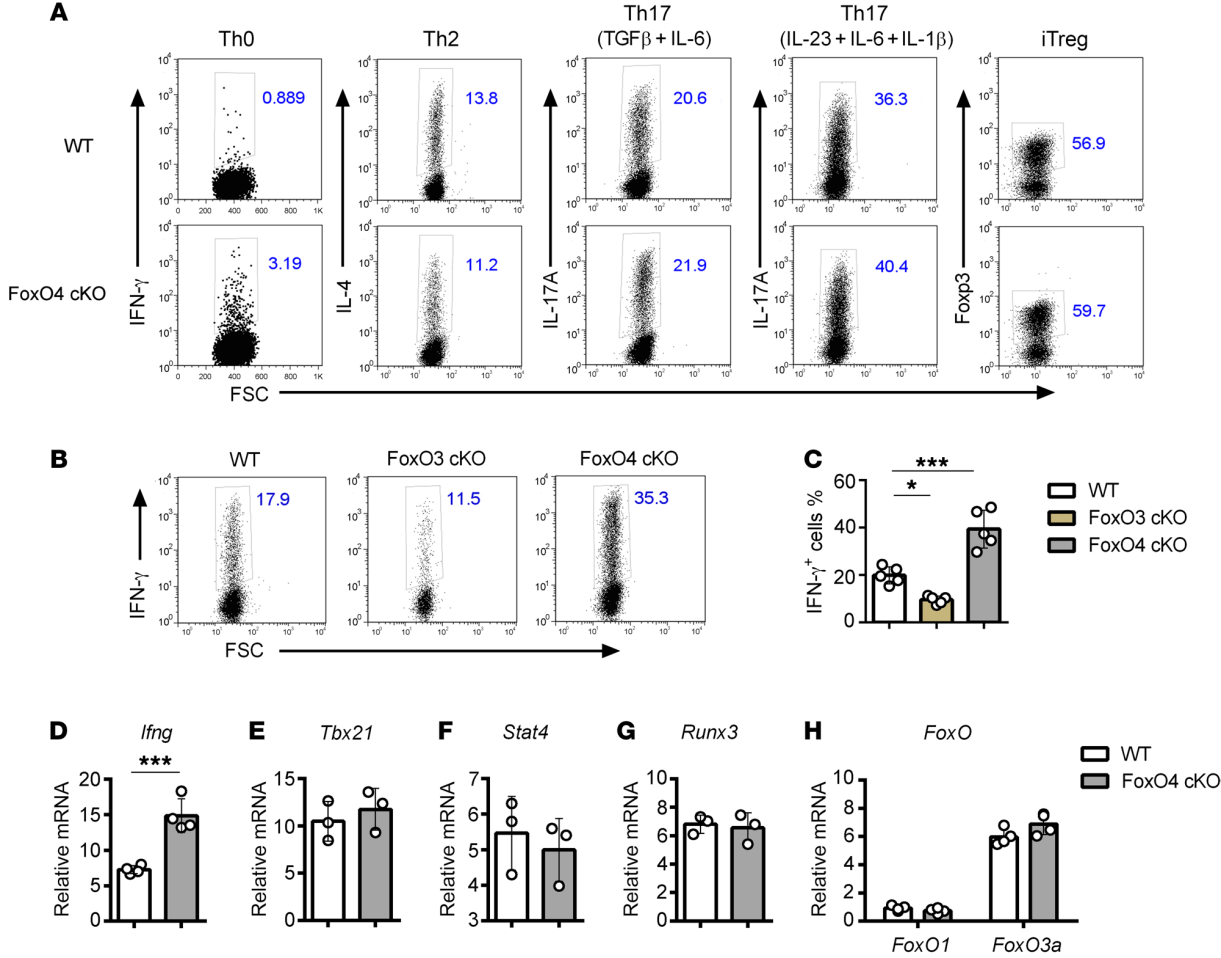
FoxO4 negatively regulates IFN-γ production in Th1 cells in vitro. (**A** and **B**) Flow cytometric analysis of naive CD4^+^ T cells after 3 days of polarization toward the Th0 (**A**), Th1 (**B**), Th2 (**A**), Th17 (**A**), and iTreg (**A**) lineages in the presence of a protein transport inhibitor in the last 5 hours (*n* = 4–5). (**B**) Flow cytometric analysis of naive CD4^+^ T cells after 3 days of polarization toward Th1 lineage, as described in **A** (*n* = 5). (**C**) Percentage of IFN-γ–expressing WT CD4^+^ T cells, *FoxO3*-cKO CD4^+^ T cells, and *FoxO4*-cKO CD4^+^ T cells (*n* = 5). Cells were gated as shown in **B**. (**D**–**H**) Real-time qPCR analysis of *Ifng* (**D**), *Tbx21* (**E**), *Stat4* (**F**), *Runx3* (**G**), and *FoxO1* and *FoxO3a* (**H**) in CD4^+^ T cells isolated from WT and *FoxO4*-cKO mice followed by stimulation for 3 days with plate-bound anti-CD3 and anti-CD28 in Th1-polarizing conditions and assessment after restimulation with plate-bound anti-CD3 for 5 hours (*n* = 3–5). Results are presented relative to the expression of *Gapdh* mRNA. *P < 0.05 and ***P < 0.001, by 1-way ANOVA with Tukey’s multiple-comparison test (**C**) or unpaired, 2-tailed Student’s *t* test (**D**–**H**). Data are representative of 3 independent experiments with similar results (mean ±SD in **C**–**H**).

We next assessed the role of FoxO4 in CD4^+^ Th cell differentiation in vitro. We sorted CD4^+^CD25^−^CD62L^hi^CD44^lo^ naive T cells by flow cytometry and differentiated them for 3–5 days in neutral (Th0), Th1, Th2, Th17, and iTreg conditions and then analyzed the lineage-specific markers by intracellular staining (Figure 2, A and B). We observed no significant difference in Th2, Th17, or iTreg differentiation. In addition, we also examined signature genes of Th2, Th17, and iTregs in corresponding skewed conditions. Consistent with the results of intracellular staining (Figure 2A and Supplemental Figure 2, A–D), real-time quantitative PCR analysis (qPCR) (Supplemental Figure 2, E–H) revealed no significant change in signature genes at the transcriptional level for these Th cell subsets. However, *FoxO4* deficiency resulted in increased IFN-γ–producing cells in the Th0 condition, in the absence of exogenous IL-12. The result indicated that FoxO4 controlled IFN-γ production independently of IL-12 signaling (Figure 2A). Since *FoxO3a* deficiency has been shown to repress Th1 differentiation, we next examined Th1 differentiation in *FoxO4*-cKO cells. Surprisingly, we found that *FoxO4* deficiency, in contrast to *FoxO3a* deficiency, enhanced Th1 differentiation (Figure 2, B and C). To further characterize *FoxO4*-deficient Th1 cells, we analyzed the expression of Th1 cell signature genes by real-time qPCR, revealing dramatically augmented levels of *Ifng* mRNA (Figure 2D). However, the expression of Th1 lineage–specific transcription factor genes, including *Tbx21*, *Runx3*, and *Stat4*, in *FoxO4*-deficient Th1 cells was comparable to that in WT cells (Figure 2, E–G), suggesting that augmented expression of IFN-γ in *FoxO4*-deficient Th1 cells was not secondary to increased expression of Th1-specific transcription factors. In addition, we evaluated endogenous IFN-γ function in both WT and *FoxO4*-cKO cells. The intracellular staining results showed elevated IFN-γ expression in *FoxO4*-cKO cells with or without anti–IFN-γ mAb treatment (Supplemental Figure 3, A and B) compared with expression in WT cells. Although anti–IFN-γ treatment substantially suppressed IFN-γ expression in KO cells, it did not completely inhibit IFN-γ expression in them. This result further supports the idea that FoxO4 negatively regulates IFN-γ expression in Th1 cells. Since the other FoxO family proteins FoxO1 and FoxO3a are also expressed and play distinct functions in Th1 cells, we suspected that FoxO4 might regulate IFN-γ through cross-regulation of FoxO family proteins. However, we did not find any difference in *FoxO1* or *FoxO3a* expression in the absence of FoxO4 (Figure 2H), which suggests that FoxO4 probably functions in Th1 cells in a nonredundant manner. Together, FoxO4 appears to play a specific and critical role in IFN-γ production in Th1 cells in vitro.

### FoxO4 deficiency enhances IFN-γ production in vivo.

**Figure 3:**
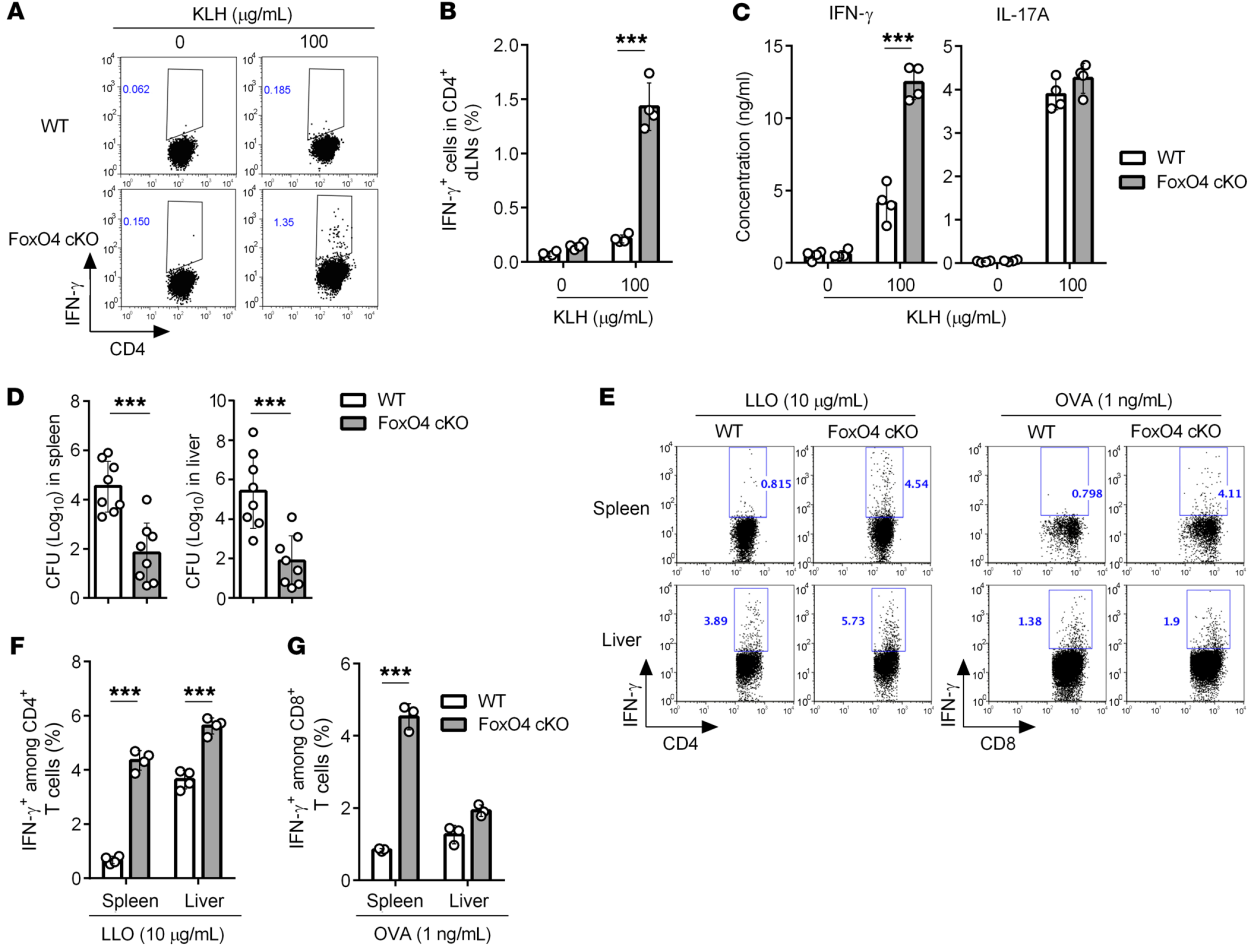
Augmented Th1 responses in vivo in the absence of FoxO4. (**A**) Intracellular staining for IFN-γ in WT and *FoxO4*-cKO CD4^+^ T cells with or without KLH stimulation (0 or 100 μg/mL) from dLNs of mice 7 days after KLH/CFA immunization (*n* = 4). (**B**) Frequency of IFN-γ–expressing cells in dLN CD4^+^ T cells as in **A** (*n* = 4). (**C**) An ELISA was performed to determine the expression of IFN-γ and IL-17A in dLNs as in **A** (*n* = 4). (**D**) *L*. *monocytogenes* titers in the spleens and livers of WT and *FoxO4*-cKO mice infected with Lm-OVA for 7 days, shown as CFU (*n* = 10). (**E**) Flow cytometric analysis of IFN-γ–producing CD4^+^ and CD8^+^ T cells in the spleen and liver as in **D**, after 5 hours of restimulation with LLO_190–201_ (left) or OVA_257–264_ (right) peptide in the presence of a protein transport inhibitor (*n* = 3–4). (**F** and **G**) Frequency of IFN-γ–expressing cells as in **E** (*n* = 3–4). ***P < 0.001, by unpaired, 2-tailed Student’s *t* test (**B**–**D**, **F**, and **G**). Data are representative of 3 independent experiments with similar results (mean ±SD in **B**–**D**, **F**, and **G**).

Having shown that *FoxO4* deficiency augmented Th1 cell differentiation in vitro, we next sought to determine whether *FoxO4*-cKO mice also produce elevated levels of IFN-γ in vivo. We immunized WT and *FoxO4*-cKO mice subcutaneously with keyhole limpet hemocyanin (KLH) in CFA to elicit a strong inflammatory response. One week after immunization, we isolated lymphocytes from draining lymph nodes (dLNs) and found germinal center (GC) B cells (B220^+^GL7^+^CD95^+^) and Tfh cells (CD4^+^CD44^hi^CXCR5^+^PD-1^+^) developed normally in *FoxO4*-cKO mice (Supplemental Figure 4, A and B), indicating that FoxO4 was dispensable for the development of Tfh cells and GC reactions. In addition, the production levels of KLH-specific antibody isotypes including IgA, IgM, IgG1, and IgG2a were similar between WT and *FoxO4*-cKO mice, (Supplemental Figure 4C). Furthermore, cell proliferation (Supplemental Figure 4D) as well as IL-2 production (Supplemental Figure 4E) in response to rechallenge with KLH were similar in CD4^+^ T cells from WT and cKO mice, indicating that FoxO4 was dispensable in regulating T cell priming and proliferation in vivo. However, intracellular cytokine staining showed that, in response to KLH restimulation, *FoxO4*-deficient CD4^+^ T cells in dLNs produced higher levels of IFN-γ (Figure 3A) with substantially increased frequencies of IFN-γ–producing CD4^+^ T cells (Figure 3B) compared with WT cells. Consistent with these results, ELISAs revealed that after restimulation with KLH, T cells from *FoxO4*-deficient mice had significantly higher expression of IFN-γ, but similar expression levels of IL-17A compared with T cells from immunized WT mice (Figure 3C), which suggested that *FoxO4* deficiency in T cells selectively altered IFN-γ production in vivo. Together, FoxO4 appeared to negatively regulate CD4^+^ T cells in their IFN-γ production in antigen-specific T cell responses in vivo.

### Loss of FoxO4 enhances Th1 cell–mediated immunity to bacterial infection.

To further investigate the role of FoxO4 in T cell–mediated antipathogen immune responses in vivo, we next applied an infection model using an intracellular bacterial pathogen, *Listeria monocytogenes*, which is known to induce strong T cell responses by the induction of IFN-γ–producing Th1 cells and CD8^+^ effector T cells. We infected age- and sex-matched WT and *FoxO4*-cKO mice using a modified Lm-OVA strain (*L.* *monocytogenes* strain expressing OVA). Seven days after infection, as expected, *FoxO4*-cKO mice showed significantly reduced bacterial burdens in the liver and spleen compared with WT mice (Figure 3D), indicating that the *FoxO4*-cKO mice were more resistant to *L.* *monocytogenes* infection.

To examine pathogen-specific CD4^+^ and CD8^+^ effector T cells during infection, we restimulated splenocytes and liver cells from infected mice with an MHC class II–restricted listeriolysin O peptide (LLO_190–201_) and an MHC class I–restricted OVA peptide OVA_257–264_ (SIINFEKL), respectively. As expected, Lm-OVA–infected *FoxO4*-cKO mice, compared with WT mice, had substantially more CD4^+^ and CD8^+^ IFN-γ–producing effector T cells following restimulation with LLO_190–201_ (Figure 3, E and F) and OVA_257–264_ (Figure 3, E and G), respectively. To determine whether the phenotypic difference in *Listeria* infection between WT and cKO mice was dependent on CD4^+^ T cells, we performed antibody-mediated depletion of CD4^+^ T cells during *Listeria* infection. Bacterial burden enumeration showed no difference in CFU in the livers or spleens of CD4-depleted WT and *FoxO4*-cKO mice, but both had higher CFU compared to normal WT and *FoxO4*-cKO mice, respectively (Supplemental Figure 5). Taken together, our findings indicate that FoxO4 serves as a negative regulator of antigen-specific Th1 cell responses to bacterial infection. Thus, loss of *FoxO4* enhanced Th1 cell–mediated immunity to bacterial infection in vivo.

### Ectopic FoxO4 expression represses IFN-γ production in Th1 cells.

**Figure 4:**
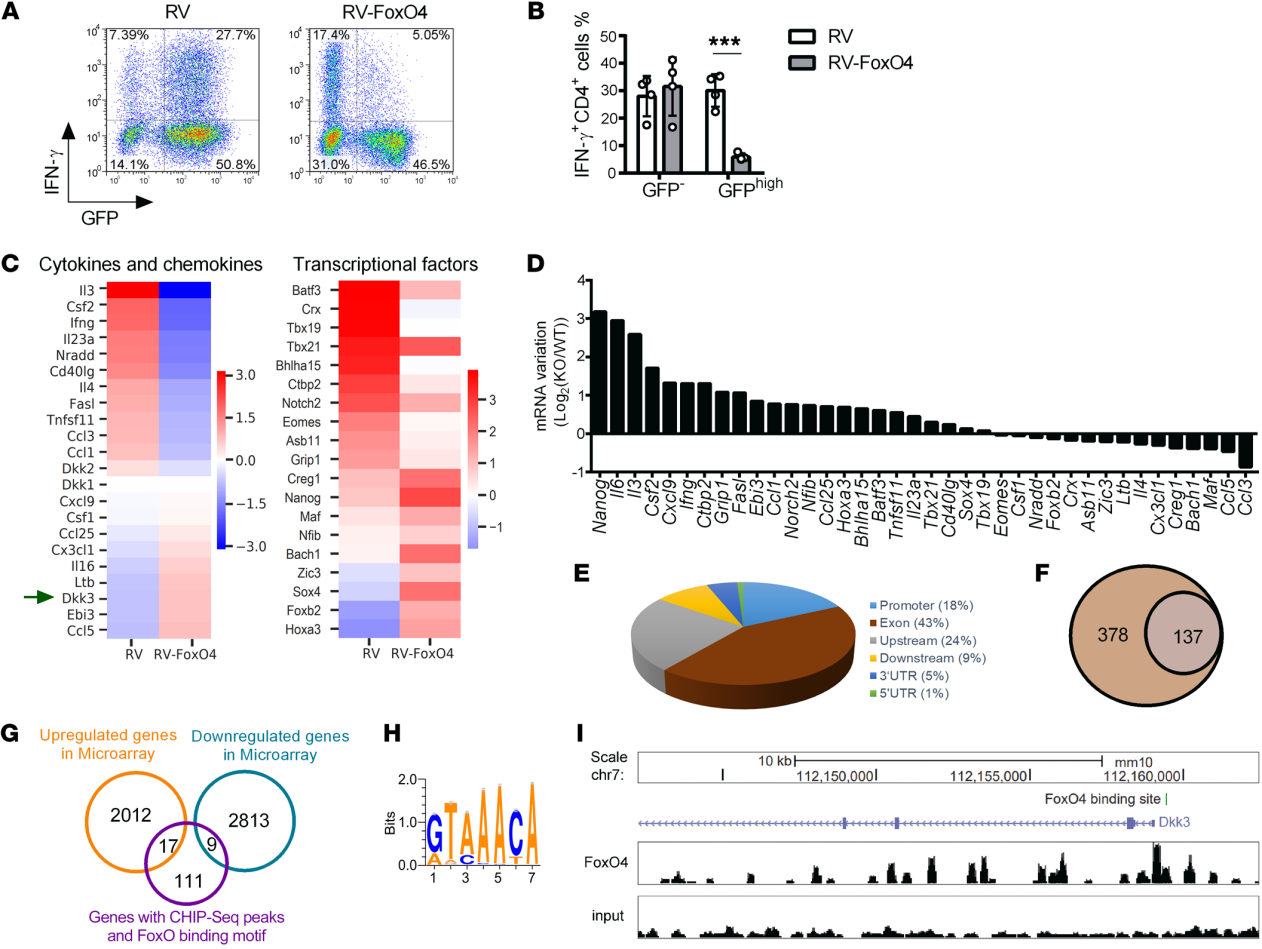
Identification of *Dkk3* as a direct target gene of FoxO4. (**A**) Flow cytometry of CD4^+^ T cells cultured under Th1-polarizing conditions and transduced with an empty vector (RV) or a vector encoding FoxO4 (RV-FoxO4), followed by restimulation of the cells and intracellular staining for IFN-γ. Data were analyzed in GFP^–^ and GFP^hi^ cell populations (*n* = 4). (**B**) Frequency of IFN-γ–expressing cells as in **A** (*n* = 4). (**C**) A gene expression microarray experiment was performed using sorted GFP^+^ cells transduced with RV or RV-FoxO4 as in **A**. Heatmap shows the gene expression intensities of some of the most significantly regulated genes (FDR <0.05 and fold change >1.5), including cytokine and chemokine genes (left) and transcriptional factor genes (right). (**D**) Real-time qPCR analysis was performed to validate gene expression changes [log_2_(KO/WT)], as in **C**, in polarized Th1 cells from WT and *FoxO4*-cKO mice after 5 hours of stimulation with plate-bound anti-CD3. (**E**) Distribution of genome-wide FoxO4-binding sites in polarized Th1 cells in vitro relative to annotated features of known genes. (**F**) Venn diagram of FoxO4-bound genes and FoxO4-bound genes containing the FoxO-binding motif. (**G**) Venn diagram of genes regulated by FoxO4 and FoxO4-bound genes containing the FoxO-binding motif. (**H**) The base sequence represents the consensus FoxO-binding motif (JASPAR), which was found at the *Dkk3* locus with a score of 11.3. (**I**) FoxO4-binding peaks located at the *Dkk3* gene locus. MACS software was applied to determine peak significance within ChIP-Seq data, and a threshold of *P* < 1 × 10^–5^ was used for peak calling. ***P < 0.001, by unpaired, 2-tailed Student’s *t* test (**B**). Data are representative of 2 independent experiments (mean ±SD in **B**).

To assess the function of FoxO4 in processes of Th1 differentiation, we activated naive CD4^+^ T cells (CD4^+^CD25^−^CD62L^hi^CD44^lo^) with plate-bound anti-CD3 and anti-CD28. Twenty hours after activation, we transduced activated CD4^+^ cells with a retrovirus expressing GFP alone (RV) or a retrovirus expressing GFP and FoxO4 (RV-FoxO4) under various T cell–skewing conditions, as described above. After infecting cells for 3 days, we assessed the cytokine production of GFP^+^ and GFP^–^ cells by intracellular cytokine staining. FoxO4 overexpression resulted in a dramatically impaired differentiation into IFN-γ–producing Th1 cells (Figure 4A) and a significantly lower frequency of IFN-γ–producing CD4^+^ T cells (Figure 4B). However, in other T cell–skewing conditions, including Th2 and Th17 cells and iTregs, no significant change was elicited by FoxO4 overexpression (Supplemental Figure 6, A and B). Thus, ectopic expression of FoxO4 specifically inhibited the differentiation of naive CD4^+^ T cells into Th1 cells.

To systematically identify genes whose expression was altered by FoxO4 overexpression during Th1 cell differentiation, we assessed global gene expression by microarray analysis in WT cells infected with both RV and RV-FoxO4 under Th1-polarizing conditions. We identified 2822 genes that were downregulated and 2029 genes that were upregulated in RV-FoxO4–infected Th1 cells relative to their expression in RV-infected Th1 cells. The analysis revealed that, among genes encoding cytokines and chemokines (Figure 4C), ectopic FoxO4 expression markedly repressed *Ifng*, *Il3*, and *Csf2* expression. In the “Transcription factors” gene expression profile (Figure 4C), we found that *Tbx21* expression in RV-FoxO4–infected Th1 cells was comparable to that in RV-infected Th1 cells, supporting our previous finding that FoxO4 did not regulate *Tbx21* expression (Figure 2E). These results were confirmed by real-time qPCR (Figure 4D). Thus, ectopic FoxO4 expression repressed IFN-γ production in Th1 cells without affecting *Tbx21* expression.

### Genome-wide analysis identifies Dkk3 as a direct transcriptional target of FoxO4.

Previous work reported that FoxO1 could negatively regulate *Ifng* transcription through binding of the *Ifng* promoter region directly (8). In addition, a recent study showed FoxO3a directly bound to the *Eomes* locus to regulate *Eomes* transcription (10), leading to an increase in IFN-γ production. However, we did not find a similar occupancy of FoxO4 by ChIP-qPCR in the *Ifng* (Supplemental Figure 7A) or *Eomes* (Supplemental Figure 7B) locus. To analyze genome-wide occupancy of FoxO4, we performed a ChIP-Seq assay and identified 515 FoxO4-binding peaks using the model-based analysis for ChIP-Seq (MACS) algorithm. The results showed extensive binding of FoxO4 at exon and transcription start site (TSS) upstream regions (Figure 4E). Next, we performed an in silico search for FoxO transcription factor–binding sites using binding profiles from the JASPAR CORE database (jaspar.genereg.net). We found that 26.6% (137 of 515) of the FoxO4 peaks contained a FoxO-binding motif and were considered to be high-confidence binding sites of FoxO4 (Figure 4F). Intersecting with the microarray data above, 17 genes containing high-confidence FoxO4-binding peaks were upregulated in RV-FoxO4–infected Th1 cells: *Arvcf*, *Cnm3*, *Dkk3*, *AB24611*, *Tmem8b*, *Gng7*, *Mam13*, *Ezh1*, *Mgat5b*, *Rsph4a*, *Dst*, *Abcg1*, *Spib*, *Tssk4*, *Trdn*, *Mypn*, and *Hrh1*. In addition, 9 genes containing high-confidence FoxO4-binding peaks were downregulated by ectopic expression of FoxO4: *Styk1*, *Cth*, *Fastkd1*, *Fosl2*, *Zfp286*, *Gnal*, *Ankrd27*, *Inmt*, and *Mboat4* (Figure 4G). Among these potential FoxO4 target genes, *Dkk3*, one member of the Dickkopf family, drew our attention, since DKK family proteins have been shown to regulate CD4^+^ T cell–mediated immune responses (24, 25). More important, the predicted binding motif for FoxO proteins (JASPAR MA0848.1) with a score of 11.3 (Figure 4H) and specific FoxO4-binding peaks were found at the *Dkk3* locus (Figure 4I). Together, these data suggested that *Dkk3* is a potential direct target of FoxO4 protein.

### DKK3 restores normal IFN-γ production in FoxO4-deficient Th1 cells through downregulating Lef1 expression.

**Figure 5:**
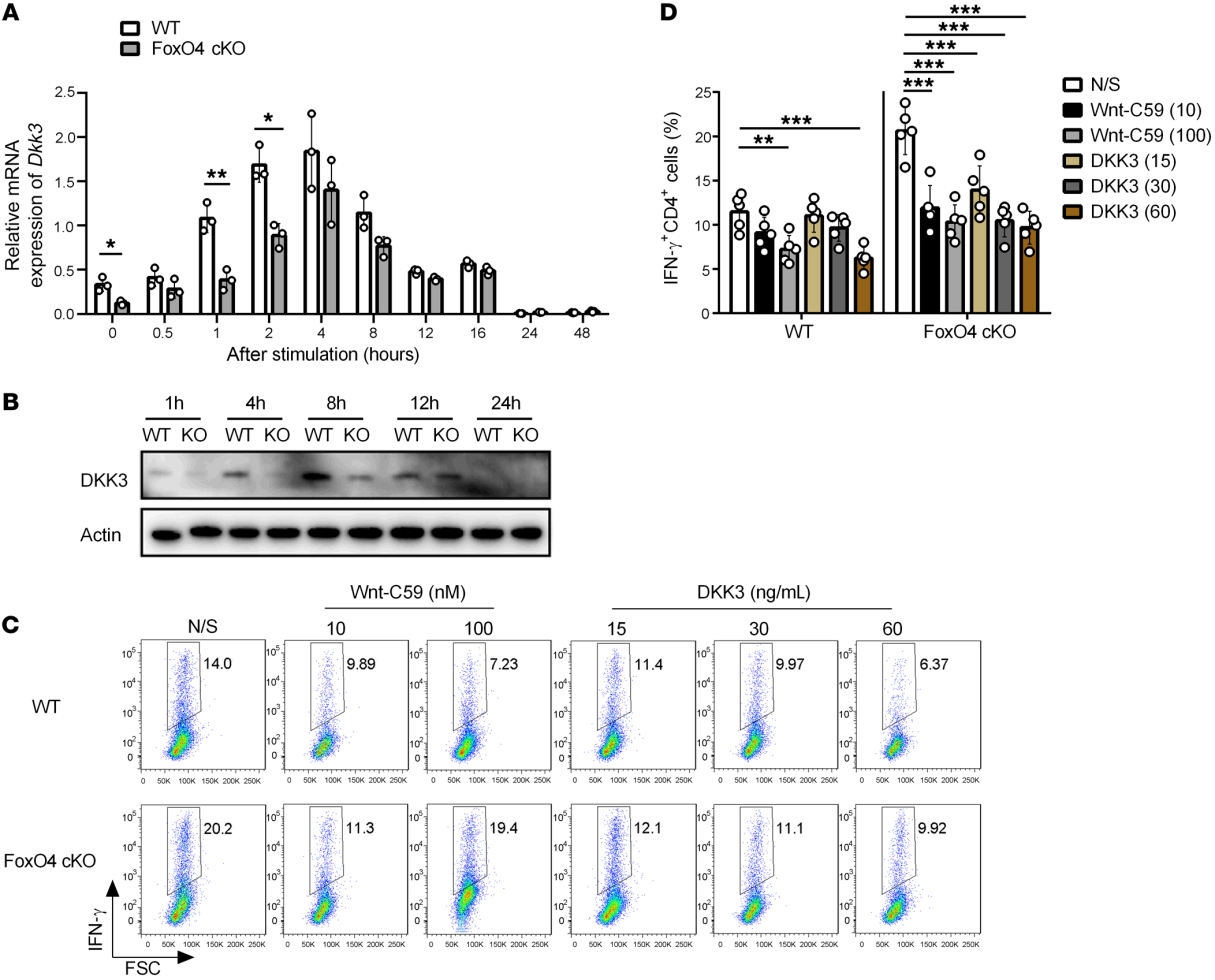
The FoxO4/DKK3 axis suppresses IFN-γ expression in Th1 cells. (**A**) Real-time qPCR analysis of *Dkk3* expression in cultured CD4^+^ T cells from WT and *FoxO4*-cKO mice in Th1-polarizing conditions at different time points (*n* = 3). (**B**) Western blot analysis of DKK3 protein expression in cultured CD4^+^ T cells from WT and *FoxO4*-cKO mice in Th1-polarizing conditions at different time points. (**C**) Intracellular staining for IFN-γ in naive CD4^+^ T cells after 2.5 days of polarization toward the Th1 lineage in the presence of Wnt-C59 (10 nM or 100 nM), a Wnt/β-catenin inhibitor, and DKK3 protein treatment (15–60 ng/mL) (*n* = 5). (**D**) Frequency of IFN-γ–expressing cells as in **C** (*n* = 5). *P < 0.05, **P < 0.01, and ***P < 0.001, by unpaired, 2-tailed Student’s *t* test (**A**), or 2-way ANOVA with Tukey’s multiple-comparison test (**D**). Data are representative of 2 (**A** and **B**) or 3 (**C** and **D**) independent experiments (mean ±SD in **A** and **D**).

Having identified *Dkk3* as potential target of FoxO4, we first examined whether *Dkk3* expression in CD4^+^ T cell is regulated by FoxO4. While we found no significant difference in *Dkk3* expression levels between WT and *FoxO4*-cKO Th1 cells 48 hours after differentiation, in a time-course analysis, *Dkk3* expression levels were indeed lower in *FoxO4*-deficient cells between 0 and 8 hours under Th1 cell differentiation conditions (Figure 5A). To further confirm that FoxO4 regulates *Dkk3* at the early stage of Th1 differentiation, we performed Western blotting to analyze DKK3 protein expression at several time points after activation under Th1-polarizing conditions (Figure 5B). Together with the qPCR data (Figure 5A), we could make a conclusion that, from 0–8 hours after activation, compared with WT cells, both mRNA and protein expression levels of *Dkk3* were decreased in *FoxO4*-KO cells, suggesting that FoxO4 played a critical role in regulating *Dkk3* transcription at the early stage of Th1 differentiation.

Next, we investigated whether treatment with DKK3 protein could overcome the inhibitory effect of FoxO4 on IFN-γ production in Th1 cells. We assessed the dose-dependent response of DKK3 on IFN-γ expression and compared it with Wnt-C59, a known Wnt/β-catenin inhibitor. Intracellular cytokine staining showed that DKK3 (50247-M08H, Sino Biological) at concentrations greater than 30 ng/mL could inhibit IFN-γ expression, similar to what we observed with Wnt-C59 (S7037, Selleck), in WT cells; however, in *FoxO4*-cKO cells, 15 ng/mL DKK3 was sufficient to override the effect of *FoxO4* deficiency on IFN-γ expression (Figure 5, C and D), indicating that DKK3 treatment could restore normal IFN-γ production in *FoxO4*-deficient Th1 cells. Next, in order to validate the function of the FoxO4/DKK3 axis in vivo, we treated WT and *FoxO4*-cKO mice with DKK3 and the Wnt inhibitor Wnt-C59, respectively, in the *Listeria* infection model. Data showed that DKK3, but not the Wnt inhibitor, significantly increased bacteria burdens in the livers and spleens of *FoxO4*-cKO mice (Supplemental Figure 8), suggesting a feedback regulation of DKK3 in *FoxO4*-deficient mice and an unclear mechanism whereby DKK3 functions in a Wnt-independent manner. Together, our current findings demonstrate that the FoxO4/DKK3 axis plays a critical role in regulating T cell immunity for *L.* *monocytogenes* infection.

**Figure 6:**
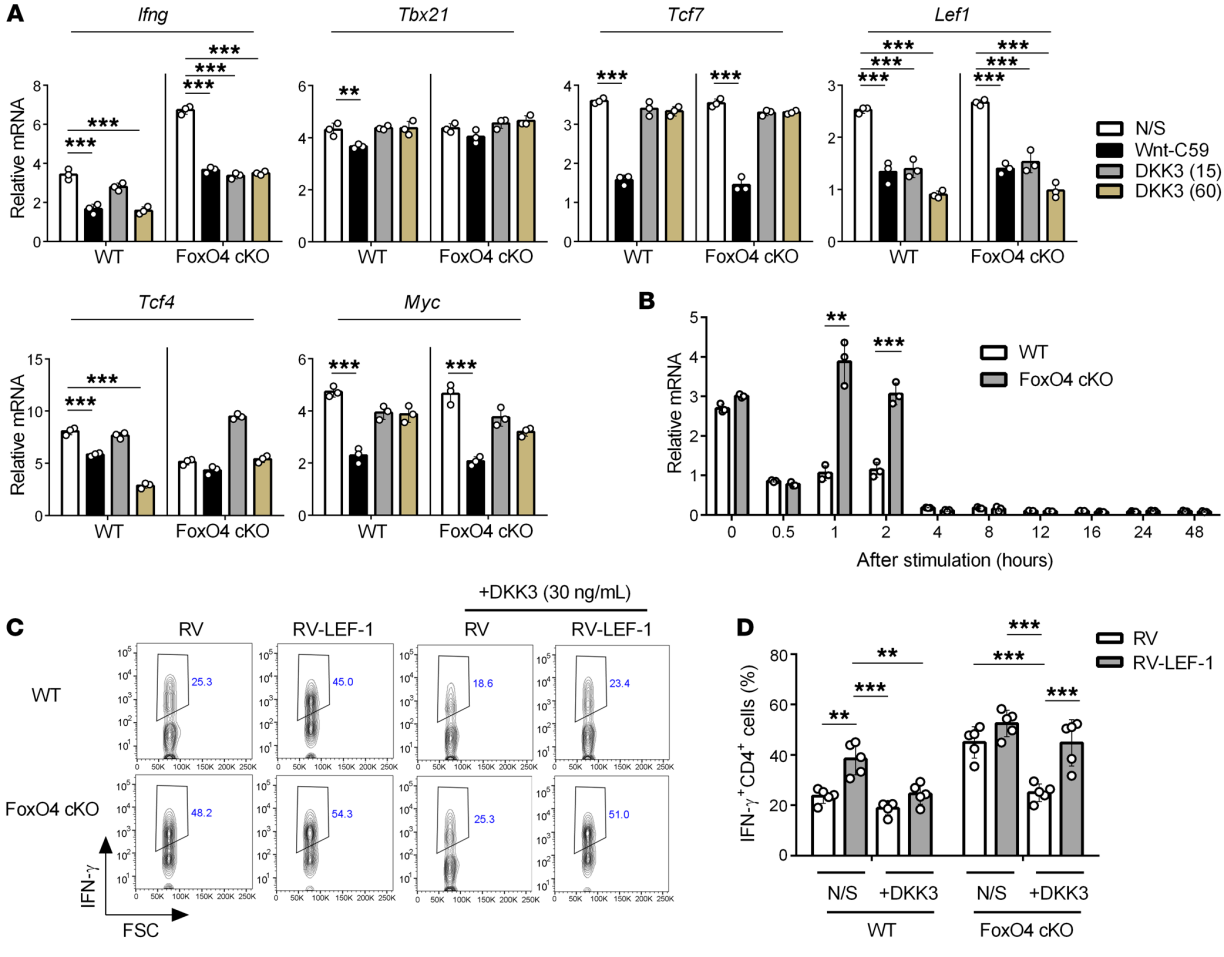
The DKK3/LEF-1 axis negatively regulates IFN-γ expression in Th1 cells. (**A**) Real-time qPCR analysis of the expression of *Ifng*, *Tbx21*, *Tcf7*, *Lef1*, *Tcf4*, and *Myc* in naive CD4^+^ T cells after 2.5 days of polarization toward the Th1 lineage in the presence of Wnt-C59 (10 nM or 100 nM), a Wnt/β-catenin inhibitor, and DKK3 protein (15–60 ng/mL) (*n* = 3). (**B**) Real-time qPCR analysis of *Lef1* expression in cultured CD4^+^ T cells from WT and *FoxO4*-cKO mice in the Th1-polarizing condition at different time points (*n* = 3). (**C**) Intracellular staining for IFN-γ in naive CD4^+^ T cells transduced with an empty vector (RV) or a vector encoding LEF-1 (RV-LEF-1) after 2.5 days of polarization toward the Th1 lineage, with or without 30 ng/mL DKK3 protein treatment (*n* = 5). (**D**) Frequency of IFN-γ–expressing cells as in **C** (*n* = 5). **P < 0.01 and ***P < 0.001, by 2-way ANOVA with Tukey’s multiple-comparison test (**A** and **D**) or unpaired, 2-tailed Student’s *t* test (**B**). Data are representative of 2 (**A** and **B**) or 3 (**C** and **D**) independent experiments (mean ±SD in **A**, **B**, and **D**).

Data published by other groups showed that β-catenin overexpression elevates the production of IFN-γ, and DKK1 promotes pathological Th2 cell–mediated inflammation (16). Therefore, we hypothesized that DKK3 could attenuate IFN-γ production by inhibiting the Wnt signaling pathway. Surprisingly, real-time qPCR results showed that, in WT cells, DKK3 treatment failed to downregulate the expression of genes relevant to the Wnt signaling pathway as broadly as Wnt-C59 did (Figure 6A). In *FoxO4*-cKO cells, we found that DKK3 treatment could attenuate the expression of *Lef1*, which encodes the transcriptional factor LEF-1, among relevant genes in the Wnt signaling pathway (Figure 6A). Although we did not find a difference in *Lef1* expression 24 hours after Th1 differentiation, it is still possible that there was a specific time period in which *Lef1* expression was different between WT and *FoxO4*-deficient Th1 cells. Thus, we assessed *Lef1* mRNA expression levels at several time points at the early stage of Th1 differentiation in WT and *FoxO4*-cKO cells (Figure 6B). We found that in the first 2 hours after T cell activation, compared with WT cells, *Lef1* expression in *FoxO4*-cKO cells was significantly increased, and then dropped to baseline levels in both cell types, suggesting that *Lef1* expression was regulated by FoxO4 at the early stage of Th1 differentiation.

Previous studies showed that *Tcf7*, which encodes the transcriptional factor T cell factor 1 (TCF-1), inhibits Th1 cell differentiation and the production of IFN-γ in a Wnt/β-catenin–independent manner (26). Moreover, *Lef1*, as another main downstream gene of the Wnt/β-catenin signaling pathway, has been shown to play a role opposite that of *Tcf7* in T cell development and malignancy (27). Therefore, it is possible that the FoxO4/DKK3 axis represses IFN-γ production by Th1 cells by regulating *Lef1* expression. To test our hypothesis, we conducted retroviral transduction with a vector encoding *Lef1* in *FoxO4*-cKO cells treated with DKK3. Intracellular cytokine staining showed that DKK3 treatment impaired IFN-γ expression in *FoxO4*-cKO cells transduced with the empty vector (Figure 6, C and D). In contrast, infection with the vector encoding *Lef1* greatly augmented IFN-γ expression in DKK3-treated *FoxO4*-cKO cells compared with *FoxO4*-cKO cells under Th1-polarizing conditions (Figure 6, C and D), indicating that ectopic *Lef1* expression in *FoxO4*-cKO cells could rescue IFN-γ expression after DKK3 treatment. In summary, *FoxO4* deficiency enhanced IFN-γ expression via downregulation of *Dkk3* and subsequent elevation of *Lef1* expression. Thus, our data demonstrate a potential FoxO4/DKK3/LEF-1 axis in the regulation of IFN-γ production by Th1 cells.

## Discussion

FoxO transcription factors regulate basic cellular processes, such as energy metabolism, stress responses, and apoptosis. In the immune system, FoxO proteins control critical molecules of T cell homeostasis and tolerance (28). Our study reveals a regulatory role of FoxO4 in Th1 cells that is different from that of FoxO1 and FoxO3a and identifies *Dkk3* as a direct transcriptional target of FoxO4 in Th1 cells. In Th1 cells, DKK3, a putative Wnt antagonist, suppressed IFN-γ production by regulating *Lef1* expression. Thus, we identified a potential FoxO4/DKK3/LEF-1 axis in the regulation of IFN-γ expression during Th1 cell differentiation, providing what we believe to be an important insight and supplement for FoxO family proteins in T lymphocyte biology. Given that our study indicates a potential regulatory axis of FoxO4/IFN-γ in Th1 cells, all 3 of the FoxO family members are likely mediators of nonredundant and specific functions in T cell biology.

To understand how FoxO4 suppresses IFN-γ expression, we explored genome-wide occupancy of FoxO4 in Th1 cells. Although we identified more than 20 molecules targeted by FoxO4, which suggested the presence of additional targets of FoxO4, we pursued an in-depth investigation of the FoxO4/DKK3 axis because of previous studies linking DKKs and CD4^+^ T cells. FoxO4 interacts broadly in Th1-associated gene loci, and *Dkk3* is one of the genes that not only interacts with FoxO4 in its gene locus but whose expression level is increased upon FoxO4 overexpression. More important, we also found a binding motif of the FoxO family protein and specific FoxO4-binding peaks at the *Dkk3* locus. Furthermore, DKK3 treatment in the *Listeria* infection model increased bacteria burdens in the livers and spleens of *FoxO4*-cKO mice. Given these findings, we believe that *Dkk3* is a — but maybe not the only — direct target gene of FoxO4.

DKK3 belongs to the Dickkopf family of proteins (DKK1–4) involved in modulating Wnt signaling pathways (14). Since Wnt/β-catenin signaling has been implicated in T cells, other regulators of the Wnt pathway determining Th1 function via regulation of IFN-γ are of interest. The Wnt antagonist family member DKK1 is known to activate serum/glucocorticoid-regulated kinase 1 (SGK-1) to promote Th2 differentiation via GATA-binding protein 3 (GATA3) (16). Tregs utilize DKK1 to regulate T cell–mediated tolerance in the T cell–mediated autoimmune colitis model (24). Another Wnt antagonist, DKK2, could suppress the cytotoxicity of NK cells in a β-catenin–independent manner (17). A previous study showed that genetic deletion or antibody-mediated neutralization of DKK3 exacerbated experimental autoimmune encephalomyelitis (EAE) with increased IFN-γ production (29). However, the direct target cells of DKK3 were not reported in this study. Surprisingly, another study has shown an opposite function of DKK3, in which DKK3 promoted Th1 cell differentiation and increased IFN-γ production indirectly via DCs (30). In our study, we found an inhibitory function of DKK3 for IFN-γ production in CD4^+^ T cells and identified FoxO4 as a regulator of DKK3. Furthermore, our data extend the function of DKK3 in T cells to that of a regulator of IFN-γ and establish DKK3 as a T cell–derived molecule that can be induced by FoxO4 activation in Th1 cells. It is currently not clear whether the FoxO4/DKK3 axis in T cells is required during autoimmunity or other infectious diseases, which warrants further investigation. In addition, we found that mRNA expression of *Dkk3* was only modestly perturbed in the *FoxO4*-cKO mice, although the protein levels were more significantly altered. We currently do not know why there was a difference in mRNA versus protein levels at the indicated time points. However, we can speculate that (a) the stability of DKK3 mRNA and protein expression could be influenced by additional epigenetic and translational regulation, and that (b) FoxO4 probably functions at the early stage of Th1 cell differentiation, which is supported by our Western blot data showing that *Foxo4*-deficient cells produced less DKK3 in a short period after stimulation. Thus, substantial mRNA expression differences, if they exist, between WT and cKO cells may be detectable in a specific, narrow time window. These possibilities may lead to more significant differences in protein, but not mRNA, expression levels in cKO cells.

Previous studies showed several different sources of DKK3, for example, CD8^+^ T cells secreting DKK3 was identified as an essential molecule for T cell tolerance (21). In addition, tissue-derived DKK3 functions as a modulator of local CD4^+^ and CD8^+^ T cell responses (29). In this study, our data suggest that CD4^+^ T cells are another potential source for functional DKK3. However, to demonstrate whether DKK3 derived from CD4^+^ T cells plays a suppressive role in Th1 cell functions in a nonredundant manner, studies using genetic strains with specific deletion of *Dkk3* in T cells may be required.

Although DKK1 and DKK2 function as Wnt antagonists, the signaling by DKK3 is still unclear, with reports showing no effect, promotion, or inhibition of the Wnt signaling pathway (18–20). Similarly, the functional roles of DKK3 in immunity are unclear, with conflicting studies reporting its immunomodulatory or immunostimulatory functions (21, 22), suggesting DKK3 may regulate immunity through a different mechanism. In our study, we found Wnt-C59, a potent Wnt inhibitor, suppressed diverse sets of genes linked to Wnt signaling in Th1 cells; however, DKK3 did not inhibit Wnt-related molecules as broadly as did the Wnt antagonist. This finding raised the possibility that DKK3 regulates IFN-γ production in Th1 cells through a Wnt-independent pathway. Although DKK3 did not inhibit Wnt-related molecules as broadly as the Wnt antagonist did, we showed that *Lef1* expression was suppressed by DKK3 in Th1 cells. Both TCF-1 and LEF-1 are downstream effectors of the Wnt signaling pathway and are essential for early T cell development (26, 27). Compared with TCF-1, LEF-1 is expressed predominantly in Th1 cells and was originally identified as a lymphoid-specific DNA-binding protein that recognizes a 5′-CTTTGAA motif in the TCRα enhancer (31). Previous studies showed that TCF-1 and LEF-1 have cooperative and opposing roles in T cell development (27), and in most cases, TCF-1 appeared to have a dominant effect (32). However, here we demonstrated a TCF1-independent function for LEF-1 in *FoxO4*-deficient Th1 cell differentiation. In our study, reconstitution of LEF-1 in *FoxO4*-deficient T cells restored DKK3-mediated IFN-γ suppression, linking the regulatory circuit of FoxO4/DKK3/LEF-1/IFN-γ.

In addition, there are several limitations in our current study. The mechanisms of DKK3 regulation of LEF-1 expression and LEF-1 promotion of IFN-γ expression remain unclear. Identification of DKK3 receptors on the cell surface may help to address these questions, which merit further study.

In conclusion, this study identified a critical role of FoxO4 in the regulation of IFN-γ production in Th1 cells. The regulatory axis of FoxO4/DKK3/LEF-1/IFN-γ was critical for host defense during acute infection. Our findings provided a basis for further investigation into how FoxO family proteins control the differentiation and function of CD4^+^ T cells and other lymphocyte lineages. Additionally, our results identified a potential target for therapeutic manipulation of acute infection and autoimmune disease.

## Methods

### Mice.

Mice with floxed *FoxO4* alleles (*FoxO4^fl/fl^*) and *FoxO3* alleles (*FoxO3^fl/fl^*) were previously generated (23) and were provided as gifts by Ronald A. DePinho (MD Anderson Cancer Center, Houston, Texas, USA). C57BL/6 and *Cd4^Cre^*-transgenic mice (B6 background) were from The Jackson Laboratory and the National Cancer Institute (NCI), NIH, respectively. Mice with T cell–specific deletion of *FoxO4* were generated by crossing *FoxO4^fl/fl^* mice with *Cd4^Cre^*-transgenic mice. The *FoxO4^fl/fl^* *Cd4^Cre^* (*FoxO4*-cKO) mice and *FoxO4^fl/fl^* (WT) littermates on the mixed background were used in the experiments. The *FoxO3^fl/fl^* *Cd4^Cre^* (*FoxO3*-cKO) mice were generated in a similar manner. All mice were maintained in specific pathogen–free animal facilities at MD Anderson Cancer Center and Tsinghua University, and all animal experiments were carried out using 6- to 10-week-old mice.

### Naive T cell stimulation and differentiation in vitro.

Naive CD4^+^CD25^−^CD62L^hi^CD44^lo^ T cells from spleens and lymph nodes were isolated using a FACSAria sorter (BD Biosciences). Purified naive T cells were stimulated with 2 μg/mL plate-bound anti-CD3 (2C11, Bio X Cell) and 2 μg/mL anti-CD28 (37.51, Bio X Cell) in the presence of 5 μg/mL anti–IFN-γ (XMG1.2, Bio X Cell), 5 μg/mL anti–IL-4 (11B11, Bio X Cell), and 40 U/mL IL-2 (Peprotech) for the generation of Th0 cells; 20 ng/mL IL-12 (Peprotech), 5 μg/mL anti–IL-4, and 40 U/mL IL-2 for the generation of Th1 cells; 10 ng/mL IL-4 (Peprotech), 10 μg/mL anti–IFN-γ and 40 U/mL IL-2 for the generation of Th2 cells; 1 ng/mL TGF-β1 (Peprotech), 10 ng/mL IL-6 (Peprotech), 5 μg/mL anti–IFN-γ, and 5 μg/mL anti–IL-4 or 10 ng/mL IL-23, 10 ng/mL IL-1β (Peprotech), 10 ng/mL IL-6, 5 μg/mL anti–IFN-γ, and 5 μg/mL anti–IL-4 for the generation of Th17 cells; and 1 ng/mL TGF-β1, 5 μg/mL anti–IFN-γ, 5 μg/mL anti–IL-4, and 40 U/mL IL-2 for the generation of iTregs. Cells were cultured in complete medium (RPMI medium containing 10% FBS, supplemented with penicillin-streptomycin, HEPES, l-glutamine, sodium pyruvate, and 2-mercaptoethanol) for 3–5 days, followed by intracellular staining and RNA preparation.

### Intracellular staining and flow cytometry.

Cells were restimulated for 5 hours with PMA (50 ng/mL; MilliporeSigma), ionomycin (500 ng/mL; MilliporeSigma), and monensin (Golgistop, BD Biosciences). After staining for cell-surface markers, intracellular staining was performed according to the manufacturer’s protocols (Cytofix/Cytoperm buffer set from BD Biosciences; Foxp3 staining buffer set from eBioscience). The flow cytometer FACSCalibur or LSRFortessa (both from BD Biosciences) and FlowJo software (Tree Star) were used for flow cytometry and data analysis. All antibodies used in this study are listed in Supplemental Table 1.

### Retroviral transduction.

FoxO4 and LEF-1 cDNA were PCR amplified and subcloned into an RVKM retroviral vector (33). Retrovirus production was carried out in Plat-E cells (ATCC) as described previously. Naive CD4^+^CD25^−^CD62L^hi^CD44^lo^ T cells were isolated and stimulated with plate-bound anti-CD3 and anti-CD28 antibodies. Twenty hours after stimulation, the supernatant with virus particles was added, and spin transduction was performed in the presence of polybrene (8 μg/mL) at 900 x *g* for 90 minutes at 30°C. Next, intracellular staining, ELISA, and real-time qPCR were carried out after 4–5 days of culturing in the appropriate Th cell–polarizing conditions. For some applications, GFP^+^ and GFP^–^ cells were sorted with a BD FACSAria.

### ELISA and real-time qPCR.

Cytokines were measured by ELISA as we described previously (33). Total RNA was extracted using TRIzol reagent (Invitrogen, Thermo Fisher Scientific). For analysis of mRNA transcripts, cDNA was generated by oligo(dT) priming and Moloney murine leukemia virus (MMLV) reverse transcriptase (Invitrogen, Thermo Fisher Scientific) and amplified in iQ SYBR Green Supermix (Bio-Rad) in the presence of specific primer pairs. Data were normalized to the *GAPDH* gene for each sample. All primer sequences used in this study are listed in Supplemental Table 2.

### KLH immunization.

Mice were immunized subcutaneously with 1 mg/mL KLH emulsified in 0.5 mg/mL CFA (100 μL per mouse). After 1 week of immunization, mice were sacrificed, and a series of analyses were performed. Briefly, KLH-specific IgA, IgM, IgG1, and IgG2a levels in serum were measured by ELISA using the SBA Clonotyping System (Southern Biotech). For intracellular cytokine staining, lymphocytes from dLNs and splenocytes were stimulated with 0 and 100 μg/mL KLH for 24 hours in the presence of monensin in the last 5 hours, and then IFN-γ– and IL-17A–producing cells were detected in the CD4^+^ cell population as described above. For analysis of cytokine expression, lymphocytes in dLNs were stimulated with 0, 5, 20, and 100 μg/mL KLH for 72 hours, followed by ELISA detection of IFN-γ and IL-17A in the supernatant.

### L. monocytogenes infection.

An erythromycin-resistant strain of Lm-OVA (recombinant *L.* *monocytogenes* expressing a truncated OVA protein, aa 134–387) was grown in brain heart infusion media supplemented with 5 μg/mL erythromycin. Age- and sex-matched WT and *FoxO4*-cKO mice were intravenously injected with 10^5^ CFU Lm-OVA in 100 μL PBS and sacrificed on day 7 after injection. The number of live bacteria lysates from infected organs was determined by measuring CFU. In brief, the livers and spleens were collected and homogenized, the homogenates were spread on brain heart infusion agar, and *L.* *monocytogenes* CFU were assessed after overnight growth at 37°C. For analysis of specific T cell responses, splenocytes and liver cells were stimulated with OVA_257–264_ (SIINFEKL) or LLO_190–201_ (NEKYAQAYPNVS) peptide overnight, in the presence of monensin in the last 5 hours, for intracellular cytokine staining or for 3 days for ELISA. For CD4^+^ T cell depletion, infected mice were treated with anti-CD4 antibody (GK1.5, Bio X Cell) intraperitoneally on days –1 and +1 respective to the challenge with Lm-OVA. Livers and spleens were harvested on day 4 after infection for enumeration of bacterial burden. For chemical treatment, prior to WT and *FoxO4*-cKO mice were infected with Lm-OVA on day 0, mice were treated with Wnt-C59 (10 mg/kg) intravenously and DKK3 (50 μg/kg) intraperitoneally, respectively, for 3 consecutive days, from day -3 to day -1. Then the bacterial burdens in spleens and livers from each group were compared on day 8.

### Microarray.

Naive T cells were activated and infected with RV and RV-FoxO4, respectively. Total RNA was extracted from sorted GFP^+^ cells, and Agilent 028005 SurePrint G3 Mouse GE 8×60K Microarrays were used to probe global gene expression changes.

### ChIP and ChIP-Seq.

The ChIP experiment was performed using Active Motif’s ChIP assay kit (catalog 53035) as we described previously (33), with anti-FoxO4 antibody (sc-5221, Santa Cruz Biotechnology) or rabbit IgG (ab37415, Abcam). The precipitated DNA was quantified by real-time qPCR with a Bio-Rad CFX96 real-time system using the primer specific for different targets. Primers for FoxO4 ChIP-qPCR were synthesized as reported previously (10).

For ChIP-Seq, input and endogenous FoxO4 ChIPed DNA obtained by the ChIP procedure above were subjected to library preparation using the NEXTflex ChIP-Seq DNA Sequencing Kit (5143, Bioo Scientific). The DNA libraries were sequenced on an Illumina HiSeq 2500 by Bionova Biotech Co. Ltd. For data analysis, the raw reads were mapped to the mm10 genome using bowtie (34), and then MACS software was used for peak calling. The motifs were called from significantly enriched peaks using the MEME suite (35). An in-house script was used to calculate the normalized and input-subtracted depth. The files were visualized in the UCSC Genome Browser. The microarray and ChIP-Seq data sets were deposited in the NCBI’s Gene Expression Omnibus (GEO) database (GEO GSE133035).

### Statistics.

All statistical analyses were performed with an unpaired, 2-tailed Student’s *t* test or 1-way or 2-way ANOVA where appropriate using GraphPad Prism 8 (GraphPad Software). A *P* value of less than 0.05 was considered statistically significant. Data are presented as the mean ± SD.

### Study approval.

All animal experiments in this study were approved by the IACUC of MD Anderson Cancer Center and Tsinghua University.

## Author contributions

XC designed and performed the experiments and wrote the manuscript. JH performed parts of the experiments, interpreted and analyzed data, and wrote the manuscript. YL, HW, YJ, FL, YC, GZ, and BSK participated in parts of the experiments. YW, XZ, HL, and QZ assisted with data analysis and interpretation. XL and XW generated the cKO mice in this study. SHC designed the experiments, supervised the study, and edited the manuscript. CD supervised the study and edited the manuscript.

## Supplementary Material

Supplemental data

## Version 1 09/15/2022

Electronic publication

## Version 2 03/13/2025

Corrected figure 6C per corrigendum
