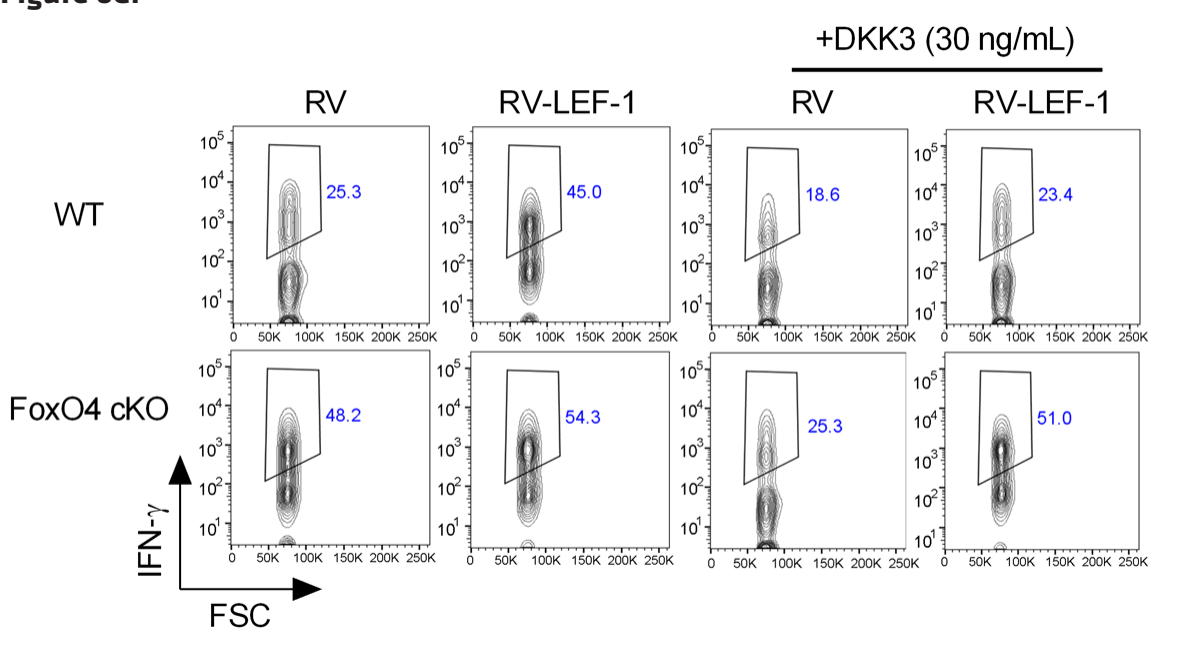# The FoxO4/DKK3 axis represses IFN-γ expression by Th1 cells and limits antimicrobial immunity

**DOI:** 10.1172/JCI191396

**Published:** 2025-03-17

**Authors:** Xiang Chen, Jia Hu, Yunfei Wang, Younghee Lee, Xiaohong Zhao, Huiping Lu, Gengzhen Zhu, Hui Wang, Yu Jiang, Fan Liu, Yongzhen Chen, Byung-Seok Kim, Qinghua Zhou, Xindong Liu, Xiaohu Wang, Seon Hee Chang, Chen Dong

Original citation: *J Clin Invest*. 2022;132(18):e147566. https://doi.org/10.1172/JCI147566

Citation for this corrigendum: *J Clin Invest*. 2025;135(6):e191396. https://doi.org/10.1172/JCI191396

In Figure 6C of the original article, there was an error in the flow cytometry contour plot for the KO-RV-DKK3 sample, which was an inadvertent duplication of the plot for the WT-RV sample. The corrected figure, based on the original source data, is provided below. The HTML and PDF versions of the paper have been updated.

The authors regret the error.

## Figures and Tables

**Figure 6C:**