# Supplementary material for: The FoxO4/DKK3 axis represses IFN-γ expression by Th1 cells and limits antimicrobial immunity
Source: J Clin Invest. 2022 Sep 15;132(18):e147566. doi: 10.1172/JCI147566 (PMC9479610; doi:10.1172/JCI147566)
Supplement: Supplemental data [file jci-132-147566-s007.pdf]

Supplemental Table 1. List of antibodies used in this study.

Antibodies	Source	Clone No.	Catalog No.
Anti-mouse CD3	BioXCell	145-2C11	BE0001-1
Anti-mouse CD28	BioXCell	37.51	BE0015-1
Anti-mouse IL-4	BioXCell	11B11	BE0045
Anti-mouse IFN-γ	BioXCell	XMG1.2	BE0055
Anti-mouse CD4	BioXCell	GK1.5	BE0003-1
Anti-mouse FoxO4	Santa Cruz	N/A	sc-5221
Anti-mouse DKK3	Proteintech	N/A	10365-1-AP
APC Anti-mouse CD4	BD Biosciences	RM4.5	553051
PerCP-Cyanine5.5 Anti-mouse CD4	eBioscience	RM4.5	45-0042-82
PE Anti-mouse CD8	eBioscience	53-6.7	12-0081-82
PE-Cyanine7 Anti-mouse CD25	eBioscience	PC61.5	25-0251-82
eFluor™ 450 Anti-mouse CD44	eBioscience	IM7	48-0441-82
APC-eFluor™ 780 Anti-mouse CD62L	eBioscience	MEL-14	47-0621-82
PE Anti-mouse IFN-γ	Biolegend	XMG1.2	505808
Alexa Fluor® 700 Anti-mouse IFN-γ	BD Biosciences	XMG1.2	557998
PE Anti-mouse IL-4	Biolegend	11B11	504104
PE Anti-mouse IL-17A	BD Biosciences	TC11-18H10	559502
FITC Anti-mouse Foxp3	eBioscience	FJK-16s	11-5773-82
APC Anti-mouse B220	BD Biosciences	RA3-6B2	553092
Alexa Fluor® 700 Anti-mouse TCRβ	BD Biosciences	H57-597	560705
FITC Anti-Mouse PD-1	eBioscience	RMP1-30	11-9981-82
Biotin Anti-Mouse CXCR5	BD Biosciences	2G8	551960

PE Anti-Mouse CD95	BD Biosciences	Jo2	554258
eFluor™ 450 Anti-Mouse GL-7	eBioscience	GL7	48-5902-82

Supplemental Table 2. Primers for Real-Time qPCR and ChIP-qPCR.

Name	Forward (5'→3')	Reverse (5'→3')
Gapdh	GACAACTTTGGCATTGTGG	ATGCAGGGATGATGTTCTG
Ifng	GATGCATTCATGAGTATTGCCAAGT	GTGGACCACTCGGATGAGCTC
Tbx21	CAACAACCCCTTTGCCAAAG	TCCCCCAAGCAGTTGACAGT
Stat4	TGGCAACAATTCTGCTTCAAAAC	GAGGTCCCTGGATAGGCATGT
Runx3	CAGGTTCAACGACCTTCGATT	GTGGTAGGTAGCCACTTGGG
Gata3	AGGGACATCCTGCGCGAACTGT	CATCTTCCGGTTTCGGGTCTGG
Il4	AGATCATCGGCATTTTGAACG	TTTGGCACATCCATCTCCG
Il5	CGCTCACCGAGCTCTGTTG	CCAATGCATAGCTGGTGATTTTT
Rorc	GACCCACACCTCACAAATTGA	AGTAGGCCACATTACACTGCT
Il17a	CTGGAGGATAACACTGTGAGAGT	TGCTGAATGGCGACGGAGTTC
Foxp3	CCCATCCCCAGGAGTCTTG	ACCATGACTAGGGGCACTGTA
Il3	GGGATACCCACCGTTTAACCA	AGGTTTACTCTCCGAAAGCTCTT
Dkk3	CTCGGGGGTATTTTGCTGTGT	TCCTCCTGAGGGTAGTTGAGA
Csf1	ATGAGCAGGAGTATTGCCAAGG	TCCATTCCCAATCATGTGGCTA
Csf2	GGCCTTGGAAGCATGTAGAGG	GGAGAACTCGTTAGAGACGACTT
Csf3	ATGGCTCAACTTTCTGCCCAG	CTGACAGTGACCAGGGGAAC
Bcl6	CCGGCACGCTAGTGATGTT	TGTCTTATGGGCTCTAAACTGCT
Ccr6	CCTGGGCAACATTATGGTGGT	CAGAACGGTAGGGTGAGGACA
Il23r	TTCAGATGGGCATGAATGTTTCT	CCAAATCCGAGCTGTTGTTCTAT
Tcf4	CGAAAAGTTCCTCCGGGTTTG	CGTAGCCGGGCTGATTCAT
Tcf7	AGCTTTCTCCACTCTACGAACA	AATCCAGAGAGATCGGGGGTC
Lef1	TGTTTATCCCATCACGGGTGG	CATGGAAGTGTCGCCTGACAG

Myc	ATGCCCCTCAACGTGAACTTC	CGCAACATAGGATGGAGAGCA
H19	GCATGGTCCTCAAATTCTGCA	GCATCTGAACGCCCCAATTA
*Ifng*CNS1	CACTTCTGTGCAACCCTTGA	AAGCACTCACTGGGTCATTG
*Ifng*CNS2	AACTGGAAAATGGCAGGCTA	CCCGAGATAAATTCCATCCA
*Ifng*CNS22	ATGACAAAATGCAGGGCTTC	CCCACACTAGATGATATATGATTTTCC
*Ifng*CNS34	AAAAGAGTCCAAGATATGAAAGCAA	GGCTTTGGGAATTCTACCTTG
*Ifng*CNS55	TGTCTCGGTGACACATCCTT	GGGAGGCAGGAGGAACTTTA
*Ifng*Promoter	CCCCACCTATCTGTCACCAT	CACCTCTCTGGCTTCCAGTT
*Eomes*FBS1	CGGGGTTTGTTTTCTTGCG	GATTGTAGGTGCCCCTTTCCT
*Eomes*FBS2	AAGATCAAGGTCTGGGAACCCG	GTGGGGAGTGTAAACAAGCCG
*Eomes*FBS3	CACCGATAAACAAGCCTCCATT	GGGTTCCCAGACCTTGATCTTTAT

Supplemental Figures

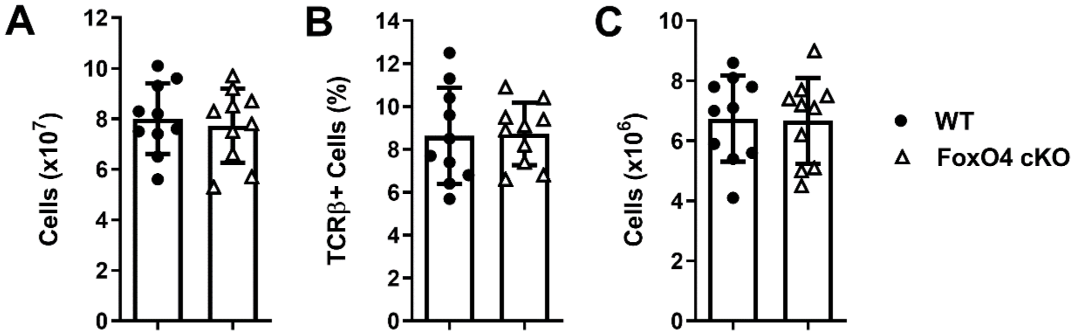

Supplemental Figure 1. Numbers of mature T lymphocytes in thymus are normal in *FoxO4* cKO mice. (**A**) Absolute thymocyte numbers in WT and *FoxO4* cKO mice (*n*=10). (**B** and **C**) Percentages (B) and absolute cell numbers (C) of TCRβ$^+$ T lymphocytes in thymus in WT and *FoxO4* cKO mice (*n*=10). Each symbol (A-C) represents an individual mouse. ns=not statistically significant, unpaired Student's *t*-test. Data are one representative of three independent experiments with similar results (means and SD in A-C).

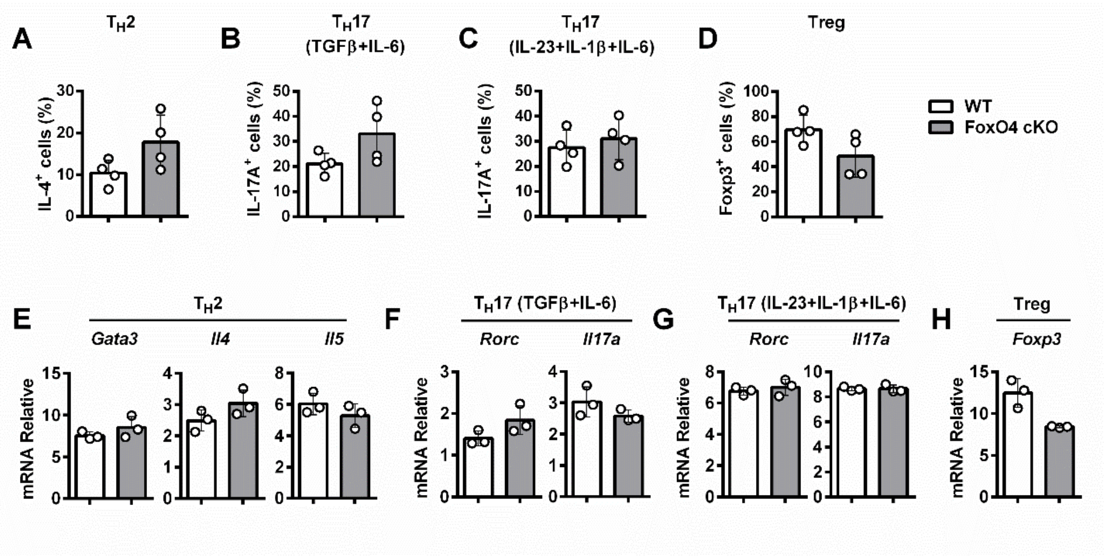

Supplemental Figure 2. FoxO4 does not affect differentiation of T_H2, T_H17, and iTreg subsets. (A-D) Percentage of IL-4 (A), IL-17A (B and C), Foxp3 (D) expressing WT and *FoxO4* cKO CD4+ T cells, which were differentiated from naïve T cells in various polarizing conditions for 3 days (n=4). (**E-H**) Real-time qPCR analysis of signature genes of T_H2 (E), T_H17 (induced by TGFβ/IL-6, F), T_H17 (induced by IL-23/IL-1β/IL-6, G) and iTreg (H) from WT and *FoxO4* cKO mice, by stimulating naive CD4+ T cells for 3 days with plate-bound anti-CD3 and anti-CD28 in various polarizing conditions, and assessing after re-stimulation by plate-bound anti-CD3 for 5 hours (n=3). Results were presented relative to the expression *Gapdh* mRNA. ns=not statistically significant, unpaired Student's *t*-test. Data are one representative of two independent experiments with similar results (means and SD in A-H).

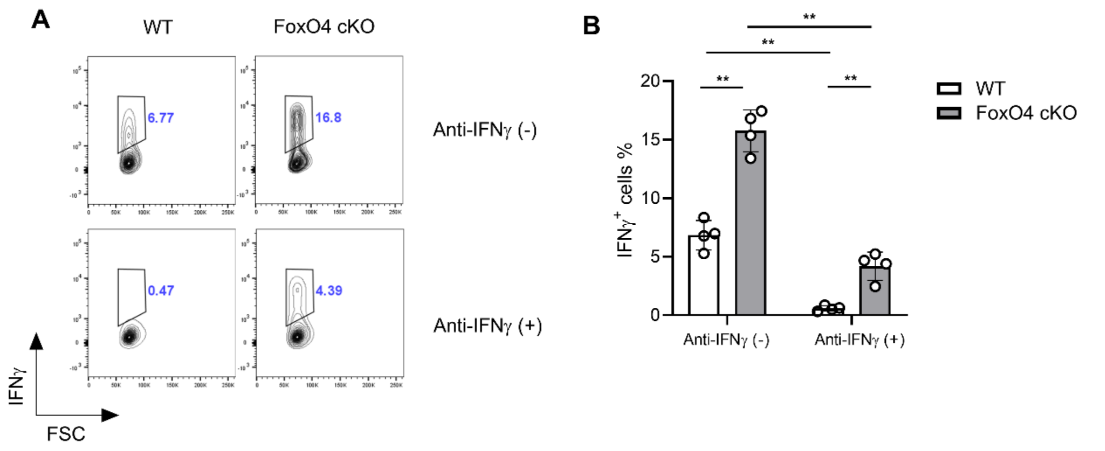

Supplemental Figure 3. FoxO4 negatively regulates IFN-γ expression in T_H1 polarizing condition with or without anti-IFN-γ. (A) Flow cytometry of WT and FoxO4 cKO Naïve CD4+ T cells after 2 days of polarization toward the T_H1 lineage, with or without anti-IFN-γ (n=4). (B) Frequencies of IFN-γ-expressing cells as in (A) (n=4). **P<0.01; ***P<0.001, by two-way ANOVA with Tukey's multiple-comparison test (B). Data are one representative of two independent experiments (means and SD in B).

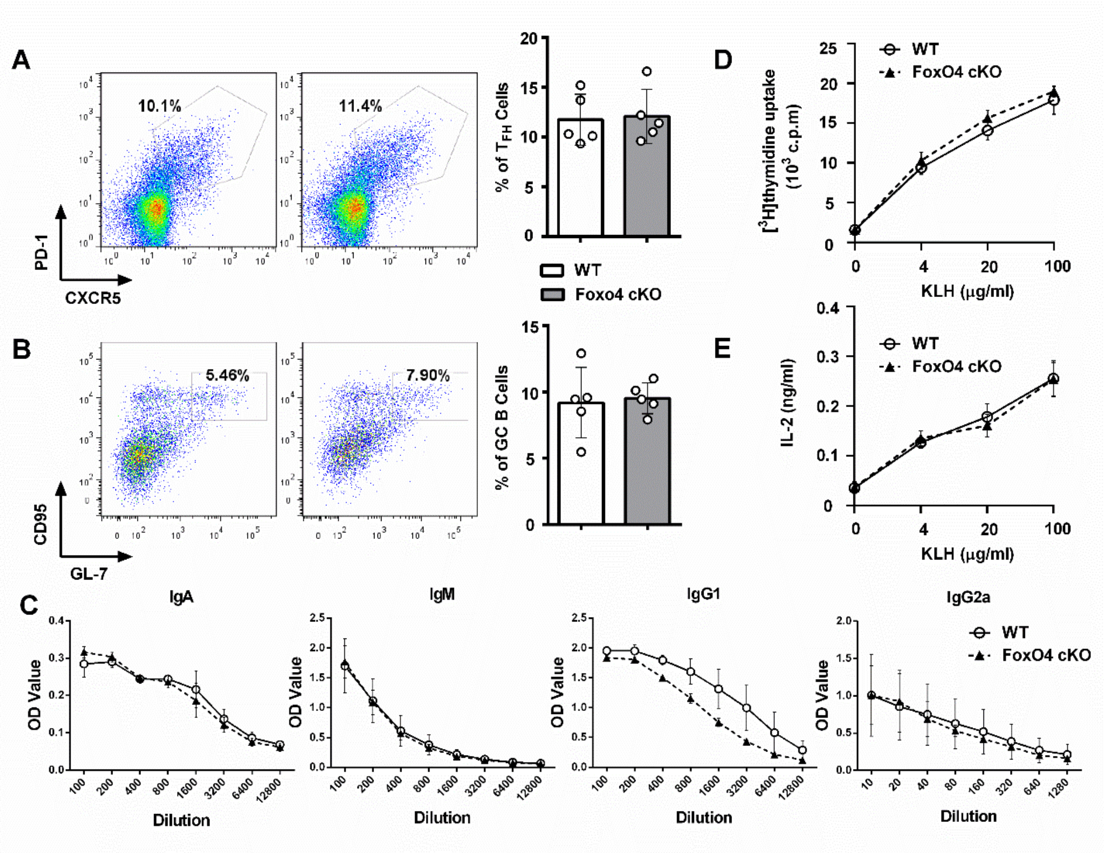

Supplemental Figure 4. *FoxO4* cKO mice have normal germinal center responses and antigen specific Ig production. (**A** and **B**) Flow cytometry analysis (left) and quantification (right) of T_{FH} cells (left in A, PD-1$^+$CXCR5$^+$, gating on CD4$^+$CD44^{hi}) and germinal center B cells (left in B, GL-7$^+$CD95$^+$, gating on B220$^+$) in dLNs from WT and *FoxO4* cKO mice which received subcutaneous KLH/CFA immunization for 7 days (n=5). (C) ELISA analysis was performed to determine the concentrations of KLH specific IgA, IgM, IgG1 and IgG2a in serum from WT and *FoxO4* cKO mice which were immunized as in A and B. Cells from draining lymph nodes were isolated 7 days later and restimulated with KLH (n=5). Proliferation was measured by [3H] thymidine incorporation assay (D) and IL-2 expression was determined by ELISA (E) (n=5). ns=not statistically significant, unpaired Student's *t*-test. Data are one representative of three independent experiments with similar results (means and SD in A-E).

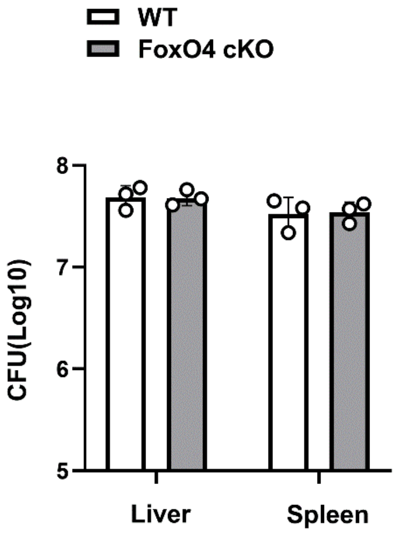

Supplemental Figure 5. FoxO4 deficiency in CD4$^+$ T cells provides protection from listeria infection. Infected mice were treated with anti-CD4 antibody (GK1.5, BioXcell) intraperitoneally at day -1 and +1 respective to challenge listeria infection. Livers and spleens were harvested at day 4 post infection for enumeration of bacterial burdens (n=3). ns=not statistically significant, unpaired Student's *t*-test. Data are one representative of two independent experiments with similar results (means and SD).

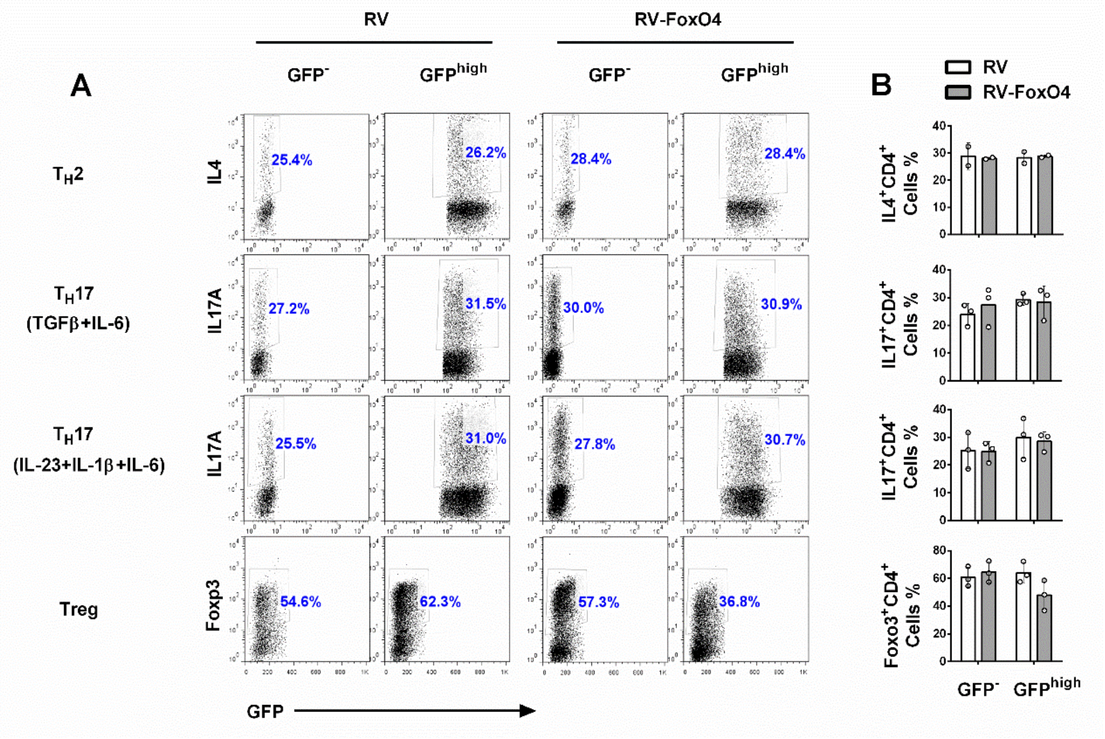

Supplemental Figure 6. Ectopic expression of FoxO4 doesn't affect differentiation of T_H2, T_H17, and iTreg subsets. (**A**) Flow cytometry of various polarized $CD4^+$ T cells, which were transduced with empty vector (RV) or vector encoding FoxO4 (RV-FoxO4). Data were analyzed in GFP^- and GFP^{high} populations, respectively (n=3). (**B**) Frequency of specific marker-expressing cells as in A (n=3). ns=not statistically significant, unpaired Student's *t*-test. Data are one representative of three independent experiments with similar results (means and SD in B).

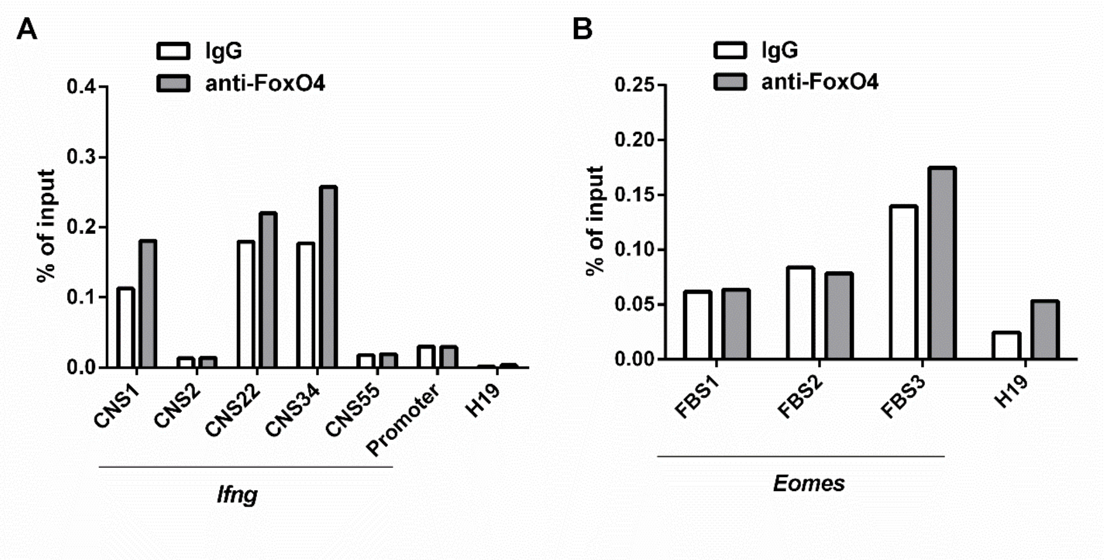

Supplemental Figure 7. FoxO4 doesn't bind to *Ifng* and *Eomes* gene loci. (**A** and **B**) Chromatin immunoprecipitation analysis of FoxO4 binding to *Ifng* (A) and *Eomes* (B) gene loci in purified $CD4^+$ T cells stimulated for 24 hours with plate-bound anti-CD3. Results were expressed as percentage of input. Data are one representative of two independent experiments with similar results.

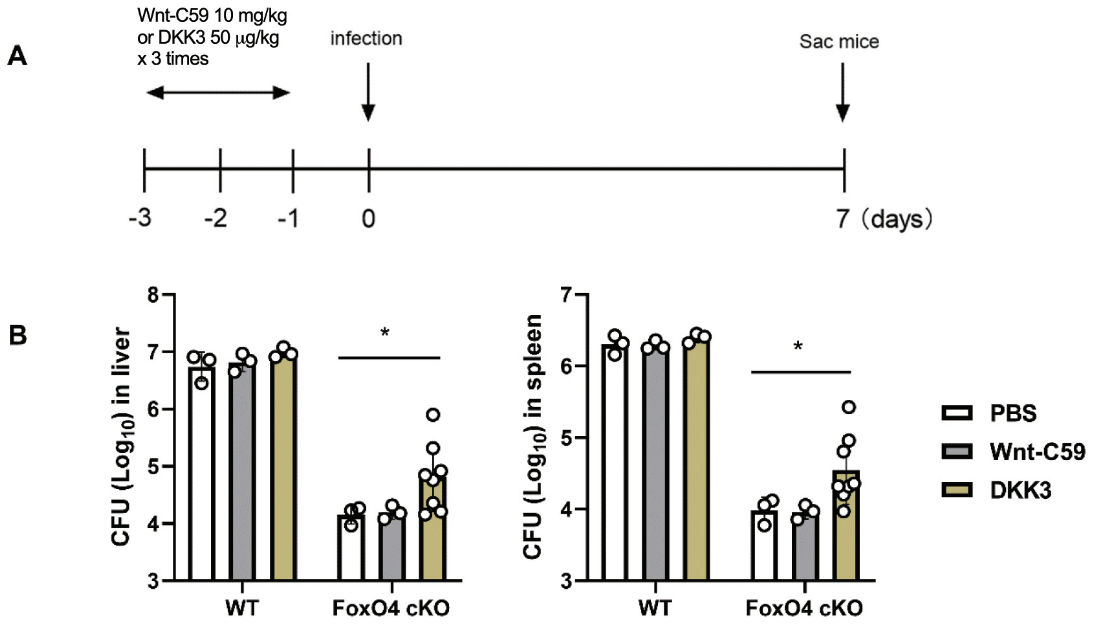

Supplemental Figure 8. FoxO4-DKK3 axis functions in anti-listeria immunity. (A) Schematic of animal experimental setup. (B) L. monocytogenes titers in the spleens and livers of WT and *FoxO4* cKO mice infected with Lm-OVA for 7 days, with treatment of PBS, Wnt-C59 and DKK3, respectively, showed as CFUs (n=3-8). *P<0.05, by two-way ANOVA with Tukey's multiple-comparison test. Data are one representative of two independent experiments with similar results (means and SD in B).

Supplemental Table 1. List of antibodies used in this study.

Antibodies	Source	Clone No.	Catalog No.
Anti-mouse CD3	BioXCell	145-2C11	BE0001-1
Anti-mouse CD28	BioXCell	37.51	BE0015-1
Anti-mouse IL-4	BioXCell	11B11	BE0045
Anti-mouse IFN-γ	BioXCell	XMG1.2	BE0055
Anti-mouse CD4	BioXCell	GK1.5	BE0003-1
Anti-mouse FoxO4	Santa Cruz	N/A	sc-5221
Anti-mouse DKK3	Proteintech	N/A	10365-1-AP
APC Anti-mouse CD4	BD Biosciences	RM4.5	553051
PerCP-Cyanine5.5 Anti-mouse CD4	eBioscience	RM4.5	45-0042-82
PE Anti-mouse CD8	eBioscience	53-6.7	12-0081-82
PE-Cyanine7 Anti-mouse CD25	eBioscience	PC61.5	25-0251-82
eFluor™ 450 Anti-mouse CD44	eBioscience	IM7	48-0441-82
APC-eFluor™ 780 Anti-mouse CD62L	eBioscience	MEL-14	47-0621-82
PE Anti-mouse IFN-γ	Biolegend	XMG1.2	505808
Alexa Fluor® 700 Anti-mouse IFN-γ	BD Biosciences	XMG1.2	557998
PE Anti-mouse IL-4	Biolegend	11B11	504104
PE Anti-mouse IL-17A	BD Biosciences	TC11-18H10	559502

FITC Anti-mouse Foxp3	eBioscience	FJK-16s	11-5773-82
APC Anti-mouse B220	BD Biosciences	RA3-6B2	553092
Alexa Fluor® 700 Anti-mouse TCRβ	BD Biosciences	H57-597	560705
FITC Anti-Mouse PD-1	eBioscience	RMP1-30	11-9981-82
Biotin Anti-Mouse CXCR5	BD Biosciences	2G8	551960
PE Anti-Mouse CD95	BD Biosciences	Jo2	554258
eFluor™ 450 Anti-Mouse GL-7	eBioscience	GL7	48-5902-82

Supplemental Table 2. Primers for Real-Time qPCR and ChIP-qPCR.

Name	Forward (5'→3')	Reverse (5'→3')
Gapdh	GACAACTTTGGCATTGTGG	ATGCAGGGATGATGTTCTG
Ifng	GATGCATTCATGAGTATTGCCAAGT	GTGGACCACTCGGATGAGCTC
Tbx21	CAACAACCCCTTTGCCAAAG	TCCCCCAAGCAGTTGACAGT
Stat4	TGGCAACAATTCTGCTTCAAAAC	GAGGTCCCTGGATAGGCATGT
Runx3	CAGGTTCAACGACCTTCGATT	GTGGTAGGTAGCCACTTGGG
Gata3	AGGGACATCCTGCGCGAACTGT	CATCTTCCGGTTTCGGGTCTGG
Il4	AGATCATCGGCATTTTGAACG	TTTGGCACATCCATCTCCG
Il5	CGCTCACCGAGCTCTGTTG	CCAATGCATAGCTGGTGATTTTT
Rorc	GACCCACACCTCACAAATTGA	AGTAGGCCACATTACACTGCT
Il17a	CTGGAGGATAACACTGTGAGAGT	TGCTGAATGGCGACGGAGTTC
Foxp3	CCCATCCCCAGGAGTCTTG	ACCATGACTAGGGGCACTGTA
Il3	GGGATACCCACCGTTTAACCA	AGGTTTACTCTCCGAAAGCTCTT
Dkk3	CTCGGGGGTATTTTGCTGTGT	TCCTCCTGAGGGTAGTTGAGA
Csf1	ATGAGCAGGAGTATTGCCAAGG	TCCATTCCCAATCATGTGGCTA
Csf2	GGCCTTGGAAGCATGTAGAGG	GGAGAACTCGTTAGAGACGACTT
Csf3	ATGGCTCAACTTTCTGCCCAG	CTGACAGTGACCAGGGGAAC
Bcl6	CCGGCACGCTAGTGATGTT	TGTCTTATGGGCTCTAAACTGCT

Ccr6	CCTGGGCAACATTATGGTGGT	CAGAACGGTAGGGTGAGGACA
Il23r	TTCAGATGGGCATGAATGTTTCT	CCAAATCCGAGCTGTTGTTCTAT
Tcf4	CGAAAAGTTCCTCCGGGTTTG	CGTAGCCGGGCTGATTCAT
Tcf7	AGCTTTCTCCACTCTACGAACA	AATCCAGAGAGATCGGGGGTC
Lef1	TGTTTATCCCATCACGGGTGG	CATGGAAGTGTCGCCTGACAG
Myc	ATGCCCCTCAACGTGAACTTC	CGCAACATAGGATGGAGAGCA
H19	GCATGGTCCTCAAATTCTGCA	GCATCTGAACGCCCCAATTA
*Ifng*CNS1	CACTTCTGTGCAACCCTTGA	AAGCACTCACTGGGTCATTG
*Ifng*CNS2	AACTGGAAAATGGCAGGCTA	CCCGAGATAAATTCCATCCA
*Ifng*CNS22	ATGACAAAATGCAGGGCTTC	CCCACACTAGATGATATATGATTTTCC
*Ifng*CNS34	AAAAGAGTCCAAGATATGAAAGCAA	GGCTTTGGGAATTCTACCTTG
*Ifng*CNS55	TGTCTCGGTGACACATCCTT	GGGAGGCAGGAGGAACTTTA
*Ifng*Promoter	CCCCACCTATCTGTCACCAT	CACCTCTCTGGCTTCCAGTT
*Eomes*FBS1	CGGGGTTTGTTTTCTTGCG	GATTGTAGGTGCCCCTTTCCT
*Eomes*FBS2	AAGATCAAGGTCTGGGAACCCG	GTGGGGAGTGTAAACAAGCCG
*Eomes*FBS3	CACCGATAAACAAGCCTCCATT	GGGTTCCCAGACCTTGATCTTTAT
